# Optimizing Availability and Appropriate Use of Assisted Vaginal Birth: Protocol for Generic Formative Research of an Implementation Preparation

**DOI:** 10.2196/69808

**Published:** 2025-09-08

**Authors:** Rana Islamiah Zahroh, Ana Pilar Betrán, Susana Lauer Betrán, Charles Kaboré, Meghan A Bohren

**Affiliations:** 1 Gender and Women’s Health Unit Nossal Institute for Global Health, School of Population and Global Health The University of Melbourne Carlton Australia; 2 UNDP-UNFPA-UNICEF-WHO-World Bank Special Programme of Research Development and Research Training in Human Reproduction (HRP) World Health Organization Geneva Switzerland; 3 Research Institute of Health Sciences Ouagadougou Burkina Faso

**Keywords:** assisted vaginal birth, ventouse, vacuum, forceps, implementation science, childbirth, labor, cesarean section, formative research, qualitative research, behavior change

## Abstract

**Background:**

Assisted vaginal birth is a lifesaving procedure where health workers use special devices to expedite birth vaginally when some complications emerge, such as due to prolonged labor. When the use of assisted vaginal birth is possible and appropriate, it provides benefits over cesarean section. These benefits include shorter recovery, reduced hospital stays, lower risks of complications, cost savings, and greater likelihood of vaginal birth in future pregnancies. Despite these benefits, the use of assisted vaginal births has declined in recent years. Challenges remain on how to implement assisted vaginal birth effectively, equitably, and at scale in different health systems. Formative research is useful to understand local health system preparedness, acceptability, feasibility, and resource availability for designing and implementing health interventions.

**Objective:**

We developed a generic formative research protocol that can be used to design and implement interventions to optimize use of assisted vaginal birth in any setting and context.

**Methods:**

This formative research protocol is to be conducted in the setting where interventions to optimize assisted vaginal birth will be implemented. The formative research has three components: (1) document review, (2) readiness assessment, and (3) primary qualitative research. Document review will examine existing policy and guidelines, while readiness assessment will assess the service delivery context. The 2 components aim to identify potential barriers and facilitators to use of assisted vaginal birth. Primary qualitative research will involve women, families, community members, health workers, facility administrators, and policy makers. Interview questions are structured around potential interventions to optimize use of assisted vaginal birth. This protocol includes all the essential study instruments.

**Results:**

This is a generic research protocol that can be used by any stakeholder aiming to optimize assisted vaginal birth in any setting. Serving as a preparatory guide, it provides a structured approach for planning and implementing evidence-based and context-specific strategies aligned with best practices in maternal health care. No data collection or analysis has been conducted to date.

**Conclusions:**

This generic protocol can serve as a guide in conducting formative research as intervention preparation to optimize use of assisted vaginal birth. The results can inform the design and implementation of appropriate interventions from the perspectives of important stakeholders. Therefore, we recommend that trialists, clinicians, researchers, administrators, and policy makers use this protocol before implementation to understand local context and incorporate perspectives of key stakeholders to promote equitable, high-quality, and women-centered care.

**International Registered Report Identifier (IRRID):**

PRR1-10.2196/69808

## Introduction

### Background

Assisted vaginal birth is a lifesaving procedure where health workers use special medical devices to help women give birth vaginally. Assisted vaginal birth instruments include forceps and vacuum extractors. Since 2009, the World Health Organization (WHO) has included assisted vaginal birth as one of the “essential signal functions” of basic and comprehensive emergency obstetric care [1]. Women may benefit from an assisted vaginal birth during the second stage of labor when challenges around prolonged labor exist [2]. These challenges may be due to the baby’s head position, fetal distress, maternal exhaustion, or medical indications that limit women’s ability to push or require speeding up the birth [2]. When the use of assisted vaginal birth is possible and appropriate, it provides advantages compared with cesarean birth [3-5]. Compared with cesarean birth, assisted vaginal birth is linked to faster recovery, shorter hospital stays, lower risk of hemorrhage and infection, cost-effectiveness, and increased likelihood of spontaneous vaginal birth in subsequent pregnancies [3-5].

However, assisted vaginal birth also has some risks, including increased likelihood of perineal tears, long-term pelvic floor dysfunction, and maternal sepsis [6-8]. Neonatal trauma, including superficial scalp or facial lacerations and cephalohematoma, can occur with assisted vaginal births [6,9]. In rare cases, more severe injuries such as brain hemorrhage, skull fractures, and nerve-related problems may also occur [2]. However, the likelihood of experiencing these adverse complications from assisted vaginal birth is exceedingly low [2,6], especially compared with the risks associated with a cesarean section at full cervical dilatation [2]. Furthermore, it is difficult to determine whether these risks are associated with assisted vaginal birth, or with the conditions that lead to the need of the intervention [2]. Mitigating these risks necessitates the careful selection of women for whom assisted vaginal birth may be appropriate, and trained and skilled professional to perform the procedure [6]. Furthermore, effective communication with women regarding the potential benefits and risks of assisted vaginal birth is crucial for informed decision-making.

Despite the potential benefits, assisted vaginal birth has decreased in recent years [10-13]. Globally, there are wide variations in assisted vaginal birth rates, ranging from 1.1% in Latin America, 1.7% in Africa, 2.5% in Asia, 3.1% in the United States, and 6.8% in Europe [10-13]. Variations are also observed within regions: in Europe, assisted vaginal birth rates exceed 12% in France, United Kingdom, Spain, and Ireland, but remain below 2.5% in Romania, Croatia, Lithuania, Slovakia, and Latvia [12,13]. Assisted vaginal birth is more common in high-income countries than in low- and middle-income countries (LMICs) [10,14]. The reasons for the low use of assisted vaginal birth are multifactorial. Women and health workers may prefer cesarean birth to assisted vaginal birth due to its perceived safety, quick action, convenience in scheduling, and when analgesia for assisted vaginal births is not available [15-17]. Limited training, equipment, supervision, and support, and fear of litigation also contributed to the low use of assisted vaginal births by health workers [18,19].

### Improving Appropriate Use of Assisted Vaginal Birth

Improving availability and quality of assisted vaginal birth may reduce maternal and neonatal morbidity and mortality without resorting to cesarean birth [2]. This is particularly beneficial in LMIC settings where many births occur in primary care facilities with limited access to timely emergency care [3,20]. In these settings, cesarean safety may not be guaranteed, and cesarean birth may require referral to a higher-level facility [3,20,21]. However, challenges remain on use of assisted vaginal birth, particularly on how it can be implemented effectively, safely, equitably, and at scale in LMICs [3]. A 2023 technical brief from WHO identified the critical gaps in optimizing assisted vaginal birth [13,22]. This included gaps in how to best educate and train health workers to acquire and maintain knowledge and skills to perform assisted vaginal birth [13]. Gaps also remain in evidence-based guidelines and protocols, equipment availability, pain relief for women, clinical and supervisory support, and the sustainability of these resources [13]. Additionally, evidence gaps exist on how to effectively inform and engage women in assisted vaginal birth decision-making [23] and to prevent misconceptions and foster informed decision-making. Given these complexities, stand-alone training interventions are likely to be insufficient to optimize use of assisted vaginal birth [13]. Instead, integration of training into multifaceted interventions to improve physiological labor management may be needed [13]. Designing such interventions should consider various factors influencing their success, which include women’s and families’ preferences and values, health worker behaviors, and health system and legal contexts. WHO recommends that programs aiming to optimize assisted vaginal birth should conduct formative research before implementation to identify and explore context-specific factors that may influence the availability and appropriate use of assisted vaginal birth [13].

We developed a generic formative research protocol to understand context-specific barriers and facilitators in the use of assisted vaginal birth. This formative research should be considered as the first stage of a multistage research project. The next stage would be to design implementation strategies to address the identified barriers and reinforce facilitators, followed by implementation and evaluation. This protocol serves as a guide and includes research instruments to assist research teams in conducting formative research in any context and setting. Throughout this protocol, we used the term “optimizing” when referring to improving the availability and appropriate use of high-quality and timely assisted vaginal birth. This term is to consider 2 different settings. First, in settings where cesarean has replaced assisted vaginal birth and is no longer practiced; therefore, reintroduction of assisted vaginal birth is needed. Second, in settings where use of assisted vaginal birth is limited and increased use may play an important role in improving outcomes.

### Objectives

The specific objectives of the formative research are to (1) explore national and health facility policies that may impact the availability and feasibility of interventions aimed at optimizing assisted vaginal birth; (2) assess the readiness of health facilities to implement any interventions aimed at optimizing use of assisted vaginal birth; (3) explore preferences, expectations, acceptability, and feasibility of the interventions aimed at optimizing use of assisted vaginal birth from the perspectives of key stakeholders: women, communities, health workers, and policy makers; and (4) explore potential factors (barriers and facilitators) that may influence intervention implementation.

## Methods

### Conceptual Framework

#### Factors Influencing the Use of Assisted Vaginal Birth

Decision-making on whether to use assisted vaginal birth is complex, occurring in emergency settings influenced by preferences, knowledge, and experiences of health workers, women, and the health care environment. Health workers may not have the knowledge and skills to decide to or to conduct assisted vaginal birth [3]. Where health workers have been trained, they may hesitate to perform assisted vaginal birth, due to the perceived complexity of assisted vaginal birth, fears of failed attempts, litigation following poor outcomes, or HIV transmission [3]. Addressing these challenges requires improving health workers’ knowledge and skills, task clarity, mentoring, task sharing, and ensuring resources and equipment availability [3]. Moreover, unsupportive work environments can hinder use, especially when clinical protocols for transitioning from assisted vaginal birth to cesarean birth are unclear [3]. Thus, clear protocols, supportive supervision, and local ownership over assisted vaginal births initiatives are critical [3].

**Figure 1:**
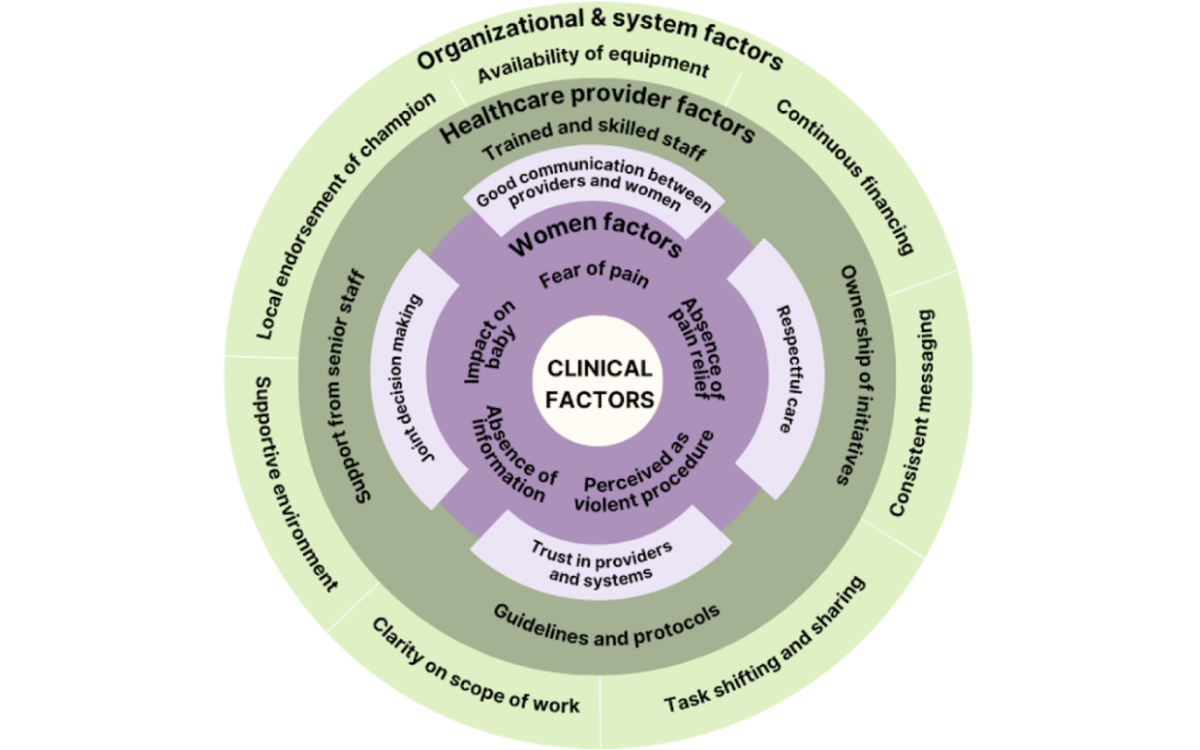
Ecological model on assisted vaginal birth decision-making. Adapted from Bohren et al [24] on factors influencing cesarean birth.

Women’s perceptions and experiences are also essential to consider [23]. Women may have fears about assisted vaginal birth due to pain relief absence, exclusion from decision-making, and lack of partner or family support [3]. Negative interactions with health workers can also hinder use, often stemming from power imbalances, poor communication, and mistrust of health systems [3]. Therefore, ensuring pain relief availability and fostering positive communication between health workers, women, and partners or families are critical. Figure 1 demonstrates the complexity of assisted vaginal birth uptake, emphasizing the importance of examining various influencers from different stakeholders before implementing any intervention.

#### Set of Interventions to Optimize Assisted Vaginal Birth

**Figure 2:**
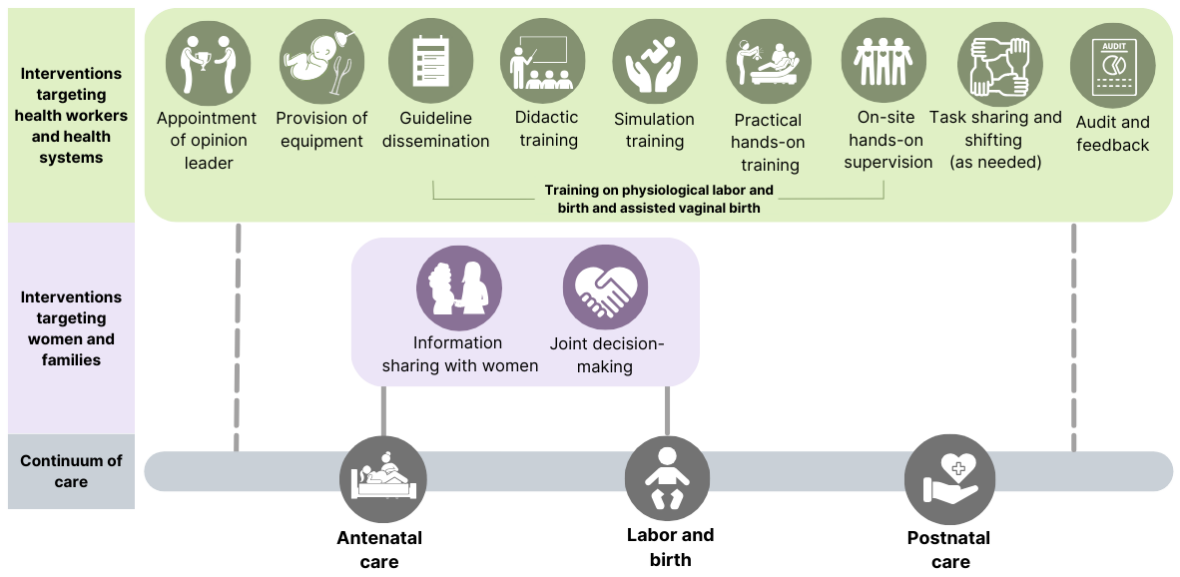
Schematic representation of potential interventions to optimize use of assisted vaginal birth.

Interventions proposed to optimize use of assisted vaginal birth are often part of broader interventions to improve labor management [3], an approach also encouraged by the WHO [13]. These may include education and training, appointing opinion leaders, providing equipment, implementing guidelines, task shifting and sharing, and audit and feedback [3]. The education and training should cover both assisted vaginal birth and broader physiological labor and birth management [3]. To date, most interventions have focused on addressing health facility and health worker factors, with little consideration of women, partners, or community views. WHO has emphasized the need to understand women’s knowledge, belief, and fears about assisted vaginal birth. Information sharing with women and joint decision-making are paramount to women’s involvement, and exploring how best to achieve this is crucial [13,23]. Figure 2 depicts the potential interventions that may be helpful to optimize use of assisted vaginal birth [3,13] In this study protocol, we provide options to explore which interventions may be suitable to address the identified facilitators and barriers in any given setting. We propose using behavior change frameworks to map barriers and facilitators identified in the formative research, which will help identify potential strategies.

#### Linking Intervention Design to Behavior Change and Implementation Science Frameworks

Growing evidence indicates that understanding and addressing individual and organizational behaviors are critical to improve implementation of evidence-based practices [25,26]. One model that captures the complexity of different behaviors is the COM-B (Capability, Opportunity, and Motivation) model of behavior change [26]. COM-B posits that individuals require capability, opportunity, and motivation to perform a desired behavior [26]. Capability points to individual capacity, including knowledge and skills, which influences the desired behavior [26]. Motivation includes drivers of the desired behavior, such as goals, emotions, beliefs, and impulses [26]. Opportunity refers to external factors outside of the individual that may influence the desired behavior [26]. Opportunity includes existing policies, communities’ perceptions, and environmental structures [26]. Both an individual’s capability and opportunity influence their motivation, which in turn affects their intended behavior [26]. Similarly, the intended behavior then impacts their overall capability, motivation, and opportunity [26]. The Theoretical Domains Framework (TDF) expands the COM-B model to categorize the barriers and facilitators to change [27]. The barriers and facilitators under the COM-B and TDF domains can be mapped to the Behavior Change Wheel to identify potential interventions that address barriers and reinforce facilitators [26]. The Behavior Change Wheel comprises 9 types of interventions and 7 policies that can be implemented to promote behavior change [26].

In this protocol, we propose the use of the COM-B and TDF frameworks to guide the study design and instruments to identify barriers and facilitators influencing the optimization of assisted vaginal birth. The “desired behavior” is defined as appropriate use of assisted vaginal birth. In addition to these frameworks, this protocol development was guided by the potential interventions identified by Torloni et al [3], the ecological framework in Figure 1, and the WHO technical brief on optimizing use of assisted vaginal birth [13].

### Study Designs and Settings

This formative research consists of three research components: (1) document review, (2) health facility readiness assessment, and (3) primary qualitative research. This formative research will be conducted in communities, health offices, and health facilities where interventions to optimize assisted vaginal birth will be implemented. The study sites will be health facilities that provide childbirth care services. These could be public and private health facilities and of different levels (tertiary, secondary, and primary), as appropriate in the study setting. Site-specific adaptations of this protocol may be necessary, which should be discussed within the research team.

### Document Review

Document review can inform methods to evaluate intervention impact, identify resources and procurement change needs, and help understand factors affecting an intervention’s sustainability postintervention [28]. The type of documents to review should be discussed with the research team and relevant stakeholders, such as health workers, government officers, and policy makers. The documents may include, but are not limited to, national guidelines, policies, professional responsibilities including reference to legal status on who and where assisted vaginal birth can be conducted, training materials, health system capacity, human resources availability, functional equipment, clinical protocols, algorithms, or decision-making charts [28]. Although some documents may be publicly available, the research team might need to contact relevant authorities to obtain them. If public or aggregate data are unavailable, the team may need to request facility-specific data. In this formative research, the document review focuses on maternity care context and information about assisted vaginal birth. The research team will review the relevant documents and enter the extracted information into a designated instrument. This document review instrument (Multimedia Appendix 1) is designed based on SUPPORT tools using evidence from local conditions [28].

### Health Facility Readiness Assessment

Following the document review, the research team will be better informed to conduct readiness assessment. Readiness assessment aims to describe and assess the service delivery context ahead of implementation. Logistically, readiness assessment can be conducted simultaneously with qualitative research and should be conducted at all study sites. The readiness assessments ensure that intervention design and implementation considerations are informed by the actual context. The readiness assessment will describe physical space, health workforce, models of care, clinical protocols, data availability, training needs, and audit and feedback practices in study health facilities. These service environment descriptions can be synthesized with primary qualitative research results to identify important barriers and facilitators to address. The readiness assessment instrument can be found in Multimedia Appendix 2.

The readiness assessment will be conducted at each health facility involved in the study. Approval will be sought from the management of the health facilities before conducting the readiness assessment. The research team will arrange meetings with facility managers to explain the readiness assessment’s purpose, scope, and methods, and request approval to proceed. Once approval is received, all relevant staff will be informed of the purpose and procedures of the readiness assessment. Since there will be no direct interaction or consultation with women, their consent will not be sought for the readiness assessment observations.

At least 1 team member with clinical knowledge will visit the health facility to observe the labor ward and relevant documents (eg, associated guidelines and clinical protocols). The readiness assessment may take several hours, depending on how busy the facility is. The readiness assessment will be scheduled with the facility managers to ensure minimal disruption to routine clinical duties. It may be helpful to conduct assessments during non–peak hours to reduce interference with clinical work. The research team will wear an identification badge and maintain a respectful distance to ensure that privacy is not compromised and to avoid encountering sensitive or clinical interactions.

### Primary Qualitative Research

This study proposes using in-depth interviews (IDIs) and focus group discussions (FGDs) to understand barriers and facilitators to the use of assisted vaginal birth use from the perspectives of women, family and community members, health workers, facility administrators, and policy makers. These perspectives encompass perceptions, preferences, experiences, acceptability, expectations, and feasibility concerning assisted vaginal birth, interventions to optimize its use, and how to best implement them. A suitable mix of stakeholder groups should be identified through consultation with the local research team, health workers, and community members.

Given the limited use of assisted vaginal birth globally, many participants may not be familiar with this procedure, making it challenging to engage meaningfully in IDI and FGD. To address this, we have developed educational materials, consisting of a comic and a frequently asked questions document (Multimedia Appendices 3 and 4), to facilitate participants’ understanding. When a participant is unfamiliar with assisted vaginal births, data collectors will first explain the frequently asked questions document and participants will be given time to review it, followed by presenting the comic to further illustrate the procedure. These materials will be delivered in the participant’s preferred language, using culturally appropriate terminology. To ensure consistency across IDI and FGD, all data collectors will be trained on how to introduce assisted vaginal birth with the comic and frequently asked questions document. This will standardize how the educational materials are introduced and explained.

The use of comics for disseminating health information to lay people has been previously used in clinical and public health settings [29-31]. Comics can be easily read and transcend barriers of literacy, language, knowledge, age, and culture [30], making them a potential tool for conveying information easily about assisted vaginal birth. To avoid triggering anxiety among participants, the comic focuses on the continuum of care, depicting the entire birth process rather than assisted vaginal birth as a stand-alone procedure. To ensure cultural acceptance of the comic and the frequently asked questions document, the research team may need to discuss and adapt to their setting.

#### Participants

The following participants are proposed for the primary qualitative research to better understand the care context:

1. Maternity service users, including
  women who gave birth in the past 6 months and experienced assisted vaginal birth;
  women who gave birth in the past 6 months and have not experienced assisted vaginal birth;
  women’s representatives who work in women’s organizations and agencies;
  women’s partners;
  mothers-in-law; and
  community members (traditional birth attendant, queen mother, village champion, older adult women, or others depending on the settings).
2. Health workers involved in antenatal care and childbirth, including
  obstetricians and other doctors who work in the maternity ward, including trainees and medical officers;
  midwives and nurses; and
  other skilled birth attendants, as appropriate.
3. Facility administrators, including
  matron-in-charge of the labor ward;
  head of obstetrics;
  medical director; and
  finance, legal, and other administrators.
4. Policy makers, including
  ministry of health staff, and
  local health office staff.

We propose to talk to women who gave birth in the past 6 months—both who *have* and *have not* experienced assisted vaginal birth in the past 6 months—and women’s representatives from women’s organizations and agencies. A 6-month postpartum time frame is set to ensure that women have sufficient recovery time while still recalling their birth experience. There will be no exclusion criteria imposed, such as clinical complications, mode of birth, intrapartum stillbirth, and so on. In settings where some degree of assisted vaginal birth is practiced, we suggest conducting IDIs with women who have assisted vaginal birth in the past 6 months, FGDs with women who have given birth in the past 6 months, and IDIs with women’s representatives. However, we acknowledge that it may be challenging to find women who underwent assisted vaginal birth in settings with limited or no use of the procedure. In this type of setting, we propose to conduct only FGDs with women who have given birth in the past 6 months and IDIs with representatives from women’s organizations and agencies.

Other than women, FGDs will also be conducted with women’s partners, family members, and community members. The selection of community member types will depend on local contexts. Partners, family members, and community members will participate in combined FGDs; however, to account for gender dynamics, we propose to conduct separate sessions for men and women. The study will also involve IDIs with health workers and facility administrators at health facilities. It may also be useful to conduct IDIs with health professional associations where appropriate, and decision should be made within the research team. Finally, IDIs will be conducted with policy makers at the ministry of health or local health offices. Relevant policy makers will be those overseeing maternal and newborn health and have experience in health policy formulation around labor management, guideline development, or resource supply and distribution. We will invite participants aged 18-49 years to participate. However, depending on the context, it may be appropriate to invite women younger than 18 years, such as in settings where adolescent pregnancy is high or young pregnant women are considered emancipated [24].

#### Sampling

Maximum variation sampling will be used [32]. Maximum variation sample encourages recruitment and sampling based on diversity and uses specified parameters to stratify the sample [24,32]. Including individuals from diverse backgrounds is critical to ensure that research findings accurately reflect the perspectives of local stakeholders. Adapting a previously developed generic formative research protocol for cesarean birth that has demonstrated feasibility and usefulness [24], we have developed a sampling grid for this study (Table 1). The sampling grid will guide the recruitment, sampling approaches, and number of participants to be recruited. However, data collection should continue until data saturation is reached [33,34]. Data saturation will be determined when no new codes or themes emerge in at least 2 consecutive interviews or focus groups. The determination of saturation will be made through regular debriefings by the qualitative lead and the research team, ensuring consistency in interpretation.

The research team should ensure diverse participant representation, such as women from urban and rural areas, with different parity, modes of birth, various ethnicities, religions, and ages. Depending on the scope of planned implementation, it may not be necessary to conduct IDIs and FGDs with women in all study facilities. If the number of facilities involved in the planned implementation is small (eg, less than 5), conducting IDIs and FGDs with women in all facilities may be appropriate. However, if the number of health facilities is large (eg, more than 5), selecting some facilities may be more feasible and appropriate. Suppose a subsample of health facilities is chosen. In that case, the selected health facilities should reflect diversity based on geographical region, urban or rural settings, level of care (eg, tertiary, secondary, and primary), and number of annual births.

IDIs with health workers should be conducted at all health facilities involved in the study, as the health facility–specific barriers and enablers may vary. Facility administrators will be sampled based on their roles and, at a minimum, should include the head of obstetrics and the matron-in-charge of maternity. A diverse group of health workers will be recruited based on gender, age, and working years. In particular, the research team will ensure a balanced representation of gender among the health workers who participate in the study. Depending on the scope of planned implementation, policy makers should be sampled from both the ministry of health and the regional or district health offices.

**Table 1:** Proposed sampling grid for primary qualitative research.

Participant groups			n (per study facility)		Types of diversity to consider
Maternity care users			5-9 IDIs^a^, 1-2 FGDs^b^		Depending on the settings, where assisted vaginal birth is high or low.
	Postpartum women who experienced assisted vaginal birth in the past 6 months	3-6 IDIs		Interviewing this women’s group may be feasible only in settings where assisted vaginal birth is practiced. In these contexts, consider recruiting women from both higher and lower-level health facilities to understand potential referral pathways. Additionally, diversity in race or ethnicity, parity, urban and rural locations, and age should be ensured to reflect diverse views.	
	Postpartum women who gave birth in the past 6 months, regardless of mode of birth	1-2 FGDs (5-10 people per group)		Consider including various birth modes—cesarean section and vaginal birth—along with diversity in race or ethnicity, parity, urban and rural locations, and age to ensure a comprehensive representation of views among participants.	
	Women representatives from women’s associations or organizations	2-3 IDIs		Women’s associations should focus on women’s health and rights or those that advocate for maternal health.	
Maternity care users’ family and community members			2-4 FGDs		Depending on the context, consider gender dynamics and diversity in community member groups.
	FGDs with women only	1-2 FGDs (5-10 people per group)		This group will include mothers-in-law of women participating in interviews or focus groups, and community members. The selection of community members (traditional birth attendants, queen mothers, village champions, older adult women, or others) will depend on the context.	
	FGDs with men only	1-2 FGDs (5-10 people per group)		This group will include partners of women participating in interviews or focus groups, and community members. The selection of community members (traditional birth attendants, queen mothers, village champions, older adult women, or others) will depend on the context.	
Health workers			6-9 IDIs		Depending on the context, diversity in terms of the level of health facilities should be considered, including both lower-level and higher-level facilities.
	Doctors	2-3 IDIs		Different types of doctors, such as obstetricians, medical officers, and doctors in training, should be included. Gender, racial or ethnic, working years, and age diversity of health workers should be considered to reflect diverse views.	
	Midwives and nurses	2-3 IDIs		Different working years, genders, races or ethnicities, and ages should be considered to reflect diverse views.	
	Administrators (eg, matron in charge)	2-3 IDIs		Different types of administrators should be considered, including clinical directors, matron-in-charge, head of obstetrics, finance, and legal officers.	
Policy makers			2-3 IDIs		Different levels of policy makers, such as the ministry of health to the regional or district health office, gender, and working years, should be considered.

^a^IDIs: in-depth interviews.

^b^FGDs: focus groups discussions.

#### Recruitment

Recruitment posters in local languages will be displayed at the health facility’s waiting area, consultation rooms, community halls, public health centers, and health offices to recruit potential women, community members, health workers, facility administrators, and policy makers. The posters will contain information about the study, eligibility criteria, and how to join the study. Depending on the settings, the study team may use the phrase “women and other pregnant people” in recruitment posters to ensure inclusivity.

Additionally, the study team will approach women who gave birth in the past 6 months during their postpartum or vaccination visits, with 1 non–health facility staff member present on-site to engage potential participants. This helps prevent power imbalances between health workers and women, which could lead to feelings of coercion in participation. Using facility records, women who indicated receiving assisted vaginal birth in the past 6 months will also be contacted to invite them to participate in the study. Conversely, the research team will approach women’s representatives, partners, family members, community members, and policy makers using snowball sampling through recommendations from gatekeepers, networks, and participating women. Health workers and administrators will be recruited at the health facility, with the research team reaching out during department meetings or via telephone or email using staff records. However, the recruitment strategy for health workers should be discussed with the maternity ward heads to decide on the best approach.

Potential participants interested in participating will be given a plain language statement (Multimedia Appendix 5), informed consent form (Multimedia Appendix 6), and time to read and decide to participate. After a few days, research team members will contact the potential participants to confirm their interest in participating and arrange an IDI or FGD at a convenient time and location. The location and time will be scheduled based on participants’ convenience and, ideally, IDIs and FGDs with women will be conducted after 4-6 weeks postpartum. The potential participants will be asked to bring the signed informed consent form to the location of the interviews. IDI and FGD guides can be found in Multimedia Appendices 7-9.

#### Qualitative Study Procedures

Before the IDIs and FGDs commence, participants will be informed again about the study in more detail through a plain language statement (Multimedia Appendix 5). If they agree, they will be asked to provide consent (written or verbal, depending on the context; Multimedia Appendix 6). Trained research team members with social science or qualitative research backgrounds, and who are not affiliated with participants’ care or workplace, will facilitate the IDIs and FGDs. The gender and age of facilitators will be considered to help participants feel comfortable; for example, FGDs with women will be facilitated by a female researcher whenever possible. FGDs will be stratified by participant type (eg, women-only groups and health workers–only groups) to reduce hierarchical dynamics. Facilitators will use strategies such as turn-taking, inviting quieter participants to speak, and cofacilitation to support inclusive discussion and reduce dominance by more vocal individuals. We propose to conduct IDIs and FGDs in person; however, if not possible, the research team may opt to conduct them on the web. IDIs and FGDs will be approximately 45-90 minutes long and audio recorded. We suggest including 5-10 women per FGD. Field notes will be recorded during data collection and used during analysis to complement transcripts.

In cases where participants are unaware of assisted vaginal birth, facilitators will use comics and frequently asked questions documents to introduce women to the procedure (Multimedia Appendices 3 and 4). Participants will be reimbursed for their time and contribution, if possible, with the amount and form determined by local customs to avoid incentivizing participation. Once IDIs and FGDs are completed, participants will not be followed up unless member checking is conducted. Member checking involves returning to participants to confirm whether their perspectives have been accurately captured during transcription and analysis [35].

#### Data Management and Quality Assurance

All research team members should undergo training with practice sessions before data collection. This includes principal investigators, social scientists, research coordinators, research assistants, transcribers, translators, admin, and other team members. The training workshop will cover project background, research designs, study tools, ethical considerations, project implementation plan, and manuals. Research monitoring will also be conducted throughout the study to ensure that issues can be addressed promptly. Research leads should communicate regularly with the research team members to address any issues promptly during data collection.

Verbatim transcription of audio recordings should occur alongside the data collection, ideally by the interviewer or facilitator, to enhance the trustworthiness of the data. Each transcription will be reviewed for data quality, themes that need further exploration, feedback for future data collection, and data saturation. During transcription, audio recordings will be deidentified by assigning pseudonyms to participants. After transcription, original audio recordings will be deleted to prevent identification. All data will be permanently deleted or destroyed after a specified retention period. Local data protection laws and requirements of study sites and stakeholders (ie, ethics committee) will determine this period. Raw and deidentified data will not be shared externally, although deidentified data may be reused in future research.

Translation may be needed if research teams comprise people with diverse language abilities. The research team will determine the appropriate timing for translation, whether during transcription or after, based on the language used during the interview. The suitability of these approaches will depend on the research team's language skills, cultural considerations, and consideration that linguistic nuances could be lost during translation.

#### Data Analysis

We suggest using a thematic analysis approach for the qualitative research, as described by Braun and Clarke [36]. We recommend starting with an analysis by participant groups to identify variations in perspectives based on their backgrounds and experiences. In the next stage, perspectives from the different groups can be compared to explore similarities and differences based on participants’ backgrounds. We suggest coding data line-by-line and assigning relevant text to specific codes. These emerging codes can be organized into hierarchical groups by grouping related codes into overarching themes and patterns. The emerging themes can then be reported narratively to report the study findings. The team will explore overarching themes across different study sites to site-specific differences that must be considered during implementation. Data analysis will be conducted separately for each participant group, with triangulation used to support in-depth understanding and to assess both convergence and divergence in perspectives. We recommend conducting data analysis simultaneously with data collection to facilitate adaptations and cross-checking, and collaboratively among research team members, incorporating expertise in qualitative research, midwifery, and obstetrics to ensure high-quality analysis. Qualitative analysis software can help manage the analysis process but is optional.

The results from thematic analysis can then be mapped to the behavioral change frameworks, COM-B and TDF. Each barrier and facilitator will be mapped to the 3 COM-B domains and 14 TDF domains to diagnose the core domain driving the behaviors. Based on the domain mapping diagnoses, the Behavioral Change Wheel linked with COM-B will be used to identify the most appropriate intervention to address this. For example, if the barriers were related to reflective motivation, training and enablement can be implemented as interventions to promote the behavior [26]. For an example of how to conduct this type of analysis, see the results from a qualitative formative study to inform intervention development for postpartum hemorrhage [37,38].

Data analysis workshops may be held to facilitate analysis, interpret findings within the local context, refine effective interventions, and discuss practical implementation strategies. The workshop findings will help inform the intervention’s development and implementation considerations. The research team will facilitate the workshop, involving all relevant stakeholders, such as health facility staff, community members, and women. The research findings can then be disseminated to community leaders and members through meetings, presentations, posters, and social media.

### Study Instruments

All study instruments are available in Multimedia Appendices 1-9. These instruments include document review and readiness assessment forms, IDIs and FGDs guides, education materials (comic and frequently asked question document), plain language statements, and consent forms. The questions in the study instruments are aligned with behavioral change framework domains to help identify barriers and facilitators related to the desired behavior.

### Researcher Reflexivity

Reflexivity refers to the research team’s awareness of their role in the project and how their assumptions, experiences, and beliefs may influence the research process [39]. This research team’s positionality can be reflected throughout the research process. Reflexivity can be practiced through team discussions, debriefing, challenging assumptions and beliefs, individual note-taking, and being transparent. We anticipate the research team to comprise people from multidisciplinary disciplines, such as clinicians, social scientists, implementation science, and maternal health researchers. Reflexive discussions can, therefore, help multidisciplinary teams to find common ground, challenge assumptions, and improve the quality of data collection and analysis.

### Research Team Composition

We recommend that the research team include individuals with diverse expertise in qualitative research, community engagement, obstetrics, midwifery, and intervention and implementation research. At a minimum, we suggest that the research team comprise (1) principal investigator: coordinate and provide leadership on day-to-day implementation, (2) social scientist lead: coordinate qualitative components and help interpret the findings’ social implications, (3) facility-level research coordinators: gatekeepers at the facility site for data collection, and (4) data collectors: conduct qualitative data collection, preferably some with clinical backgrounds.

### Ethical Considerations

Ethics approval has been received from the WHO Ethics Review Committee (A66054) and the WHO Human Reproduction Programme Review Panel on Research Projects (Multimedia Appendices 10 and 11). In addition, ethics approval will be obtained in each specific setting before implementing this formative research. All participants will provide written informed consent prior to data collection. Participation is voluntary, and participants may withdraw at any time without any negative consequences. No deception will be used. Confidentiality will be strictly maintained: all data will be deidentified before analysis, stored securely, and accessible only to authorized members of the research team. Any quotations included in publications will be anonymized. No financial compensation will be provided to participants; however, reimbursement for transport or other minimal expenses may be offered where appropriate in line with local ethics approval. The contact of the local research team will be available for participants in case they need more information or require any assistance from the team.

## Results

This is a generic research protocol that can be used by any stakeholder aiming to optimize assisted vaginal birth in any setting. Its generic nature allows for application across various health care contexts, irrespective of resource availability or system complexity. It is important to note that this protocol serves as a preparatory guide, and no data collection or analysis had been initiated at the time this protocol was published. Its purpose is to provide a structured approach for clinicians, researchers, or health policy makers to planning and implementing strategies to increase, reintroduce, or enhance assisted vaginal birth practices, ensuring that interventions are evidence-based, context-specific, and aligned with best practices in maternal health care.

Aligned with the ecological framework guiding this study, the expected outputs include a comprehensive understanding of factors at the women, health workers, organizational, and system levels that influence the use of assisted vaginal birth. These findings will be mapped into the COM-B and the TDF frameworks to inform the development of context-specific, acceptable, and feasible interventions to optimize appropriate use of assisted vaginal birth. For example, if a key barrier identified is a lack of health workers’ skills in performing assisted vaginal birth, this would point to the need for targeted educational interventions, such as hands-on training, to address the gap. On the other hand, if the research identifies a lack of functioning equipment as a significant barrier, the strategy will need to focus on improving supply chains, ensuring equipment availability and maintenance, and addressing system-level logistics. This approach draws on implementation science principles to ensure that interventions are evidence-based, theory-informed, and responsive to local contexts. This protocol development was funded on November 1, 2021, by UNDP/UNFPA/UNICEF/WHO/World Bank Special Programme of Research, Development and Research Training in Human Reproduction (Human Reproduction Programme), Department of Reproductive Health and Research, World Health Organization.

## Discussion

Assisted vaginal birth is not an innovation. Having been around for over a century, various instruments have been evaluated for their success in achieving vaginal births and their impact on women and babies [40]. Implementing assisted vaginal birth requires not only physical instruments availability but also skills, competence, and expertise to conduct the procedure safely [13,41]. In addition, views and decisions around assisted vaginal birth are strongly shaped by sociocultural factors, including family, community, health workers, and the broader health care system [18,19]. Understanding influences on use of assisted vaginal birth through formative research is essential to ensure that interventions can be implemented effectively. This protocol aims to provide guidance in conducting formative research to design and implement the most appropriate interventions to optimize use of assisted vaginal birth. Conducting formative research ensures that women’s and stakeholders' voices are considered in intervention development, prioritizing equality and rights during implementation.

The formative research described in this protocol is expected to generate in-depth understanding and actionable insights into the behavioral, cultural, and systemic barriers and facilitators of assisted vaginal birth use in the settings of interest. By exploring perspectives across all the key stakeholders, the study will identify key points where change is possible within labor management practices to optimize appropriate use of assisted vaginal birth. These findings will inform the design of tailored interventions, whether educational, organizational, or policy-based, that aim not only to optimize appropriate assisted vaginal birth use but also to promote respectful and women-centered care. For example, improving health workers’ skills and confidence in assisted vaginal birth may shift clinical norms away from default reliance on cesarean section. At the same time, addressing women’s concerns and preferences through culturally sensitive materials and counseling on assisted vaginal birth can strengthen shared decision-making during labor. Ultimately, the study will contribute to the broader challenge of implementing complex interventions in dynamic health systems, where both technical skill and women-centered care matter deeply. The insights generated will support the development of interventions that are both acceptable and feasible, grounded in the realities of the settings in which they are implemented.

All women have rights to high-quality and respectful care during childbirth, yet the underuse of lifesaving procedures, such as assisted vaginal birth, takes these rights from women. Optimizing the use of assisted vaginal birth can not only save women’s and babies’ lives but also ensure the protection of women’s rights during childbirth. Furthermore, as the “essential signal function” presents the least coverage among the 9 basic and comprehensive emergency obstetric care functions [1], prioritizing improvements in quality care on assisted vaginal birth is essential. In an era where assisted vaginal birth is almost nonexistent, it is equally important to ensure that “high-quality” assisted vaginal birth can be delivered.

Ensuring assisted vaginal birth is accessible to all women who need it, regardless of gender, ethnicity, race, socioeconomic status, or geographical location, is paramount to achieving the equitable core of high-quality care and fulfillment of human rights [41]. Despite this, appropriate use of assisted vaginal birth varies globally, with high-income countries having higher prevalence than LMICs [10,12,13]. In LMICs, health workers may resort to cesarean birth without considering an assisted vaginal birth, even for women in whom it may be appropriate. This may be due to a lack of skills, difficulty, or delay in referrals, which may jeopardize the lives of women and babies. Therefore, it is essential to understand and address the challenges related to appropriate use of assisted vaginal birth in LMICs to ensure that it is available to women who need it.

Providing assisted vaginal birth while considering women’s views and needs is crucial to achieving high-quality care. However, gender inequality and power imbalances between women and health workers often strip women of their rights to actively participate in their care. During emergency situations, women often reported feeling uninformed and unprepared about the interventions they received [19,42]. This may increase women receiving procedures they do not desire or not receiving the procedure they need. This also resulted in women’s perceived negativity toward their birth experience more holistically [19,43]. Power dynamics between women, their partners, or families can also affect a woman’s autonomy regarding assisted vaginal birth. If a woman’s partner or family is unsupportive or hesitant about the procedure, it can prevent the woman from receiving the necessary procedure when required. Ensuring women are informed about assisted vaginal birth—understanding what it is, when it is needed, and its availability—is pivotal for optimizing care and outcomes. Therefore, this protocol emphasizes that providing assisted vaginal birth while considering women’s views and needs is crucial for achieving high-quality care.

Finally, given that most people giving birth identify as women, the term “women” is used throughout this protocol. However, the protocol aims to be inclusive of all gender-diverse individuals who give birth, regardless of their gender identities. We recognize that the term “women” may exclude nonbinary or gender-diverse people. Nevertheless, we emphasize that all women and gender-diverse people giving birth may experience marginalization, and all deserve respectful maternity care and consideration.

## Appendix

Multimedia Appendix 1 Document review instrument.

Multimedia Appendix 2 Readiness assessment instrument.

Multimedia Appendix 3 Assisted vaginal birth comics.

Multimedia Appendix 4 Frequently asked questions about assisted vaginal birth.

Multimedia Appendix 5 Plain language statement.

Multimedia Appendix 6 Informed consent form.

Multimedia Appendix 7 Instruments for interview or focus group discussion with maternity care users, their family, and community members.

Multimedia Appendix 8 Instruments for interview with health workers.

Multimedia Appendix 9 Instruments for interview with policy makers.

Multimedia Appendix 10 World Health Organization ERC approval.

Multimedia Appendix 11 World Health Organization RP2 approval.

## Abbreviations

COM-B: Capability, Opportunity, and Motivation
FGD: focus group discussion
IDI: in-depth interview
LMICs: low- and middle-income countries
TDF: Theoretical Domains Framework
WHO: World Health Organization

## Data Availability

This published article and its supplementary information files include all data generated or analyzed during this study.
