# Supplementary material for: Optimizing Availability and Appropriate Use of Assisted Vaginal Birth: Protocol for Generic Formative Research of an Implementation Preparation
Source: JMIR Res Protoc. 2025 Sep 8;14:e69808. doi: 10.2196/69808 (PMC12455161; doi:10.2196/69808)
Supplement: Multimedia Appendix 1 [file resprot_v14i1e69808_app1.docx]

# **Document Review**

This is the template of document review the research team can use to assess local context, through existing policy or guideline documents, that may serve as barriers and facilitators to optimise assisted vaginal birth use, including influence on the sustainability of the intervention, in a structured and systematic way. The research team should conduct discussions to decide the documents which should be included in the document review. We recommend that this may include but is not limited to national and sub-national guidelines, policies, documents listing professional responsibilities, training, capacity of the health system, availability of human resources and functional equipment, clinical algorithms or decision-making charts, and characteristics of the obstetric population. We suggest document review be conducted by research team members with a clinical or maternal health background. This template was adapted from *Bohren MA, Opiyo N, Kingdon C, Downe S, Betrán AP. Optimising the use of caesarean section: a generic formative research protocol for implementation preparation. Reproductive Health. 2019 Nov 19;16(1):170.*

## **Part 1.** Policy or strategic plans document review.

### **1.1.** National-level and sub-national regulations that legislate the availability and implementation of assisted vaginal birth.

*Please identify any relevant national and sub-national (i.e., provincial, district, regional, state) policy or strategic plan documents, including any legal, regulatory framework, or guidance that legislates the availability and implementation of assisted vaginal birth. If this is absent, please identify any relevant maternal health policy, or a section of the policy about the mode of birth (i.e., caesarean section, including caesarean on maternal request, vaginal birth after caesarean section (VBAC)). Review each of these documents and complete the table below to describe (please add more rows as necessary):*

| **Document name** | **Year of release/publication** | **Report author, commissioner or administering body** | **Any mention of assisted vaginal birth? Please specify, i.e. "Yes, assisted vaginal birth”, “No, but there is on caesarean section”** | **Supporting text related to assisted vaginal birth or if not available, other mode of birth** |
| --- | --- | --- | --- | --- |
|  |  |  |  |  |
|  |  |  |  |  |
|  |  |  |  |  |
|  |  |  |  |  |
|  |  |  |  |  |
|  |  |  |  |  |
|  |  |  |  |  |
|  |  |  |  |  |
|  |  |  |  |  |
|  |  |  |  |  |

*Other modes of birth include caesarean section, caesarean section by maternal request, and vaginal birth after caesarean section (VBAC).

### **1.2.** Strategies to improve maternal and newborn health or quality of maternity services related to assisted vaginal birth.

*Please identify any relevant national and sub-national (i.e., provincial, district, regional, state) frameworks or strategies to improve maternal and newborn health or quality of maternity services and identify where and how assisted vaginal birth is included. Review each of these documents and complete the table below to describe (please add more rows as necessary):*

| **Document name** | **Year of release/publication** | **Report author, commissioner or administering body** | **Any mention of assisted vaginal birth**  **(yes or no)** | **Supporting text related to assisted vaginal birth** |
| --- | --- | --- | --- | --- |
|  |  |  |  |  |
|  |  |  |  |  |
|  |  |  |  |  |
|  |  |  |  |  |
|  |  |  |  |  |
|  |  |  |  |  |
|  |  |  |  |  |
|  |  |  |  |  |
|  |  |  |  |  |
|  |  |  |  |  |

## **Part 2.** Clinical documents review at the health facility level.

### **2.1.** Facility data on mode of birth: unassisted vaginal birth, assisted vaginal birth and caesarean section.

*Please describe facility data on mode of birth, which include data on unassisted vaginal birth, assisted vaginal birth and caesarean section. Unassisted vaginal birth means that a forceps or vacuum were not used but other interventions (e.g. oxytocin for induction or augmentation) might be used. Please specify if no such data exists or is not regularly reported. Please add more rows or columns as needed.*

|  | | | | | | |  |
| --- | --- | --- | --- | --- | --- | --- | --- |
| **Facility name** | **City, state** | **Sector (public/private)** | **Time period of audit*** | **Total number of unassisted vaginal birth**** | **Total number of assisted vaginal birth**** | **Total number of caesarean section**** | **Total number of births** |
| [row will be for per facility] |  |  |  |  |  |  |  |
|  |  |  |  |  |  |  |  |
|  |  |  |  |  |  |  |  |
|  |  |  |  |  |  |  |  |
|  |  |  |  |  |  |  |  |
|  |  |  |  |  |  |  |  |
|  |  |  |  |  |  |  |  |
|  |  |  |  |  |  |  |  |
|  |  |  |  |  |  |  |  |
|  |  |  |  |  |  |  |  |

### *The latest data available, if possible the last 12 months.

**If raw numbers are not available or accessible, percentage (%) can be recorded

### **2.2.** Cost of birth.

*Please provide a description of the financial costs for an unassisted vaginal birth, assisted vaginal birth, and caesarean section, to the woman and her family as well as to the health care system if publicly provided, or the insurance system. Unassisted vaginal birth means that a forceps or vacuum were not used but other interventions (e.g. oxytocin for induction or augmentation) might be used. One table corresponds to a single facility data. Please duplicate tables as needed or create separate form for each facility.*

| **Audit data on mode of birth** | | | |
| --- | --- | --- | --- |
| **Cost** | **To women and family**  **(out of pocket cost for birth expense)** | **To healthcare system (public)** | **To insurance system** |
| **Unassisted vaginal birth** |  |  |  |
| **Assisted vaginal birth** |  |  |  |
| **Caesarean section** |  |  |  |

### **2.3.** Healthcare provider remuneration for assisting birth, by mode of birth.

*Please describe how much money the hospital/doctor/nurse/midwife will receive for an unassisted vaginal birth, assisted vaginal birth, and caesarean section. Unassisted vaginal birth means that a forceps or vacuum were not used but other interventions (e.g. oxytocin for induction or augmentation) might be used. One table corresponds to a single facility data. Please duplicate tables as needed or create separate form for each facility.*

|  | | **Renumeration** | | | |  |
| --- | --- | --- | --- | --- | --- | --- |
| **Cost** | **Hospital** | | **Obstetrician** | **Doctor** | **Midwife** | **Nurse** |
| **Unassisted vaginal birth** |  | |  |  |  |  |
| **Assisted vaginal birth** |  | |  |  |  |  |
| **Caesarean section** |  | |  |  |  |  |

## **Part 3.** Document review of the information given to women at antenatal care at the health facility level.

### **3.1.** Pamphlets on birth, what to expect, what not to expect, including different modes of birth explanation.

*Please summarise any information shared to women through pamphlet on birth in regards to what to expect and what not to expect during birth, as well explanation on different mode of birth (unassisted vaginal birth, assisted vaginal birth, and caesarean section; unassisted vaginal birth means that a forceps or vacuum were not used but other interventions (e.g. oxytocin for induction or augmentation) might be used). One table corresponds to a single facility data. Please duplicate tables as needed or create separate forms for each facility:*

|  |
| --- |

### **3.2.** Women’s rights during birth.

*Please summarise any information shared to women through pamphlet or posters on women’s rights during birth, such as labour companionship, joint decision making, etc. Any obscure legal documents mentioning women’s rights that is not shared to women is not included. One table corresponds to a single facility data. Please duplicate tables as needed or create separate forms for each facility:*

|  |
| --- |
