# Supplementary material for: Optimizing Availability and Appropriate Use of Assisted Vaginal Birth: Protocol for Generic Formative Research of an Implementation Preparation
Source: JMIR Res Protoc. 2025 Sep 8;14:e69808. doi: 10.2196/69808 (PMC12455161; doi:10.2196/69808)
Supplement: Multimedia Appendix 2 [file resprot_v14i1e69808_app2.docx]

# **Readiness Assessment**

A readiness assessment is conducted to describe and assess the service delivery context ahead of implementation. The results can be then integrated into implementation plans to ensure that intervention designs are informed by the real context of the settings There are five different components of readiness assessment that should be assessed:

1. Availability of operating theatre, anaesthesia, analgesia, and anaesthetists.
2. Maternity health workforce and model of care.
3. Clinical protocols and guidelines for managing clinical care during labour and childbirth and complications.
4. Understanding of labour companionship in practice.
5. The supply chain for assisted vaginal birth.

The readiness assessment should be conducted at each of the health facilities involved in the study. One team member who has some clinical knowledge should visit the facility directly to observe the labour ward and relevant documents (associated guidelines, protocols, etc). The observation may take four to six hours depending on how busy the facility is. Prior to conducting the readiness assessment, all maternity units in the facility should be informed and briefed on the readiness assessment purpose and how it will be conducted. This briefing is imperative to ensure that research activities in the facility are welcomed by the staff.

The following readiness assessment template was adapted from *Bohren MA, Opiyo N, Kingdon C, Downe S, Betrán AP. Optimising the use of caesarean section: a generic formative research protocol for implementation preparation. Reproductive Health. 2019 Nov 19;16(1):170.*

## **Part 1.** Availability of operating theatre, anaesthesia, analgesia, and anaesthetists.

### **1.1.** Description of the operating theatres (how many, general theatres/reserved for obstetrics).

*Please observe if there are any operating theatres in the facility and if there are specific operating theatres for maternity care. Please report the number of each operating theatre:*

| General operating theatre |
| --- |
| [example: the health facility has total of 3 general operating theatres] |
| Operating theatre for maternity care |
| [example: there is only one operating theatre specifically for maternity care at the facility] |

### **1.2.** Description of anaesthesia, anaesthetists, and analgesia.

*Please identify if there are anaesthesia and analgesia that women can receive for unassisted vaginal birth and assisted vaginal birth and if there are available anaesthetists that can administer them to women. Unassisted vaginal birth means that a forceps or vacuum were not used but other interventions (e.g. oxytocin for induction or augmentation) might be used.*

| Availability and options for anaesthesia |
| --- |
| [example: local anaesthesia is administered to women receiving assisted but not unassisted vaginal birth] |
| Availability and options for analgesia |
| [example: analgesia is available for women receiving assisted but not unassisted vaginal birth. Pethilorfan is normally used for women receiving assisted vaginal birth] |
| Anaesthetists’ availability and work patterns |
| [example: anaesthesia is not available at the facility on weekdays from 7 am to 8 pm] |

## **Part 2.** Health workforce and model of care.

*Completing this section may require both observation of the labour ward and a discussion with staff, such as a matron-in-charge or head of obstetrics.*

### **2.1.** Description of the health workforce number by cadre of healthcare providers (obstetricians/consultants, non-specialist doctors working in maternity, midwives, maternity nurses, anaesthetists, etc.).

|  |
| --- |

### **2.2.** Description of the model of maternity care (i.e., midwife-led, obstetrics-led).

|  |
| --- |

### **2.3.** Description of structure of shifts for each cadre of maternity staff (obstetricians/consultants, non-specialist doctors working in maternity, midwives, maternity nurses, anaesthetists, etc.)

|  |
| --- |

## **Part 3.** Protocols and guidelines for managing clinical care during labour and childbirth.

*Completing this section may require both observation of the labour ward (i.e., posters or signs) and a discussion with staff, such as a matron-in-charge or head of obstetrics.*

### **3.1.** Description of any clinical protocols or guidelines for managing *routine labour and childbirth care*. Please state the year of release/publication of the protocols or guidelines.

|  |
| --- |

### **3.2.** Description of how any clinical protocols or guidelines for managing *routine labour and childbirth* care were developed or adapted, and updated? How are these clinical protocols/guidelines used? Please state the year of release/publication of the protocols or guidelines.

|  |
| --- |

### **3.3.** Content on clinical protocols or guidelines for procedures in managing complications during labour and childbirth.

*Please identify any relevant clinical protocols or guidelines used in the facility for managing labour and childbirth complications that is relevant to caesarean section, assisted vaginal birth, and prolonged second stage of labour. Review each of these documents and complete the table below to describe (please add more rows as necessary):*

| **Document name** | **Year of publication/release** | **Report author, commissioner or administering body** | **Supporting text related to clinical care during labour and childbirth** |
| --- | --- | --- | --- |
|  |  |  |  |
|  |  |  |  |
|  |  |  |  |
|  |  |  |  |
|  |  |  |  |
|  |  |  |  |

### **3.4.** Description of how any protocols or guidelines for managing *complications during labour or childbirth care were* developed or adapted, and updated? How are these clinical protocols/guidelines used?

|  |
| --- |

## **Part 4.** Understanding of labour companionship in practice

*Completing this section may require both observation of the labour ward and a discussion with staff, e.g.: a matron-in-charge or head of obstetrics. If companionship is not currently allowed at the facility, please specify below.*

### **4.1.** Description of who is currently allowed to act as a companion for the woman.

|  |
| --- |

### **4.2.** Description of for what periods of time companionship is offered (e.g.: from admission to discharge, during labour but not childbirth, only at childbirth).

|  |
| --- |

### **4.3.** Description of the roles that companions usually undertake (e.g.: emotional support, providing food/water/tea to the woman, supporting staff).

|  |
| --- |

### **4.4.** Description of how staff currently interact with companions (confirm this with facility staff).

|  |
| --- |

### **4.5.** Existence and content of any orientation materials, protocols, or guidelines related to how staff should work with companions, or on the role of companions. If no materials exist, please state this.

|  |
| --- |

## **Part 5.** The supply chain for assisted vaginal birth.

*Completing this section may require both observation of the labour ward and a discussion with staff, e.g.: a matron-in-charge or head of obstetrics. If companionship is not currently allowed at the facility, please specify below.*

### **5.1.** Description of how the supply chain for assisted vaginal birth instruments are maintained or challenges to keep the instruments in a functioning state.

|  |
| --- |

### Any other feedback, observations, or reflections.

|  |
| --- |
