# Supplementary material for: Optimizing Availability and Appropriate Use of Assisted Vaginal Birth: Protocol for Generic Formative Research of an Implementation Preparation
Source: JMIR Res Protoc. 2025 Sep 8;14:e69808. doi: 10.2196/69808 (PMC12455161; doi:10.2196/69808)

What is assisted vaginal birth?

Comics to explain to women and families about assisted vaginal birth and the instruments that are used in the context of research

Study protocol: Optimising availability and appropriate use of assisted vaginal birth: a generic formative research protocol for implementation preparation

Artist: Susana Lauer Betrán

Comics depicting vacuum use

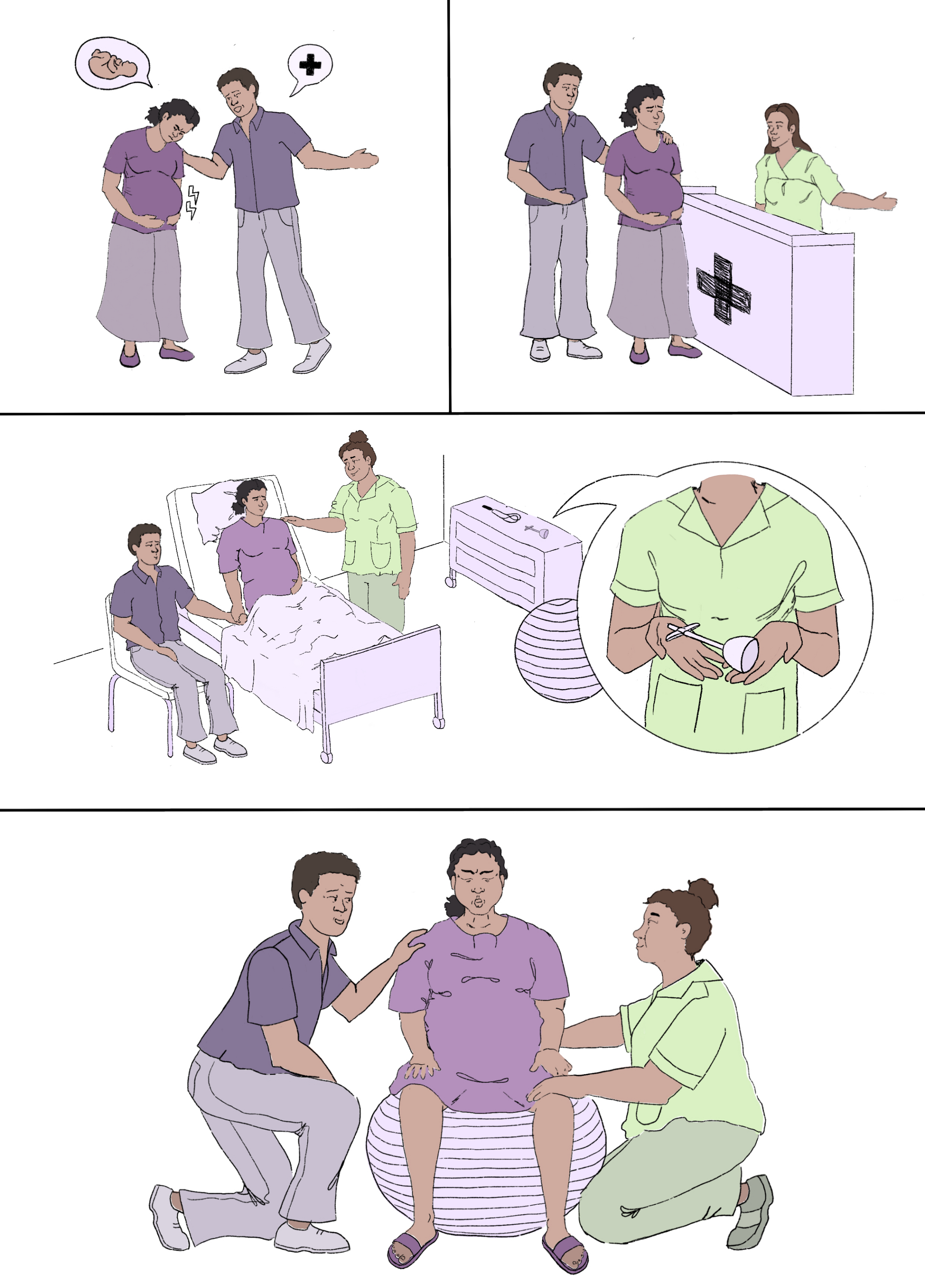

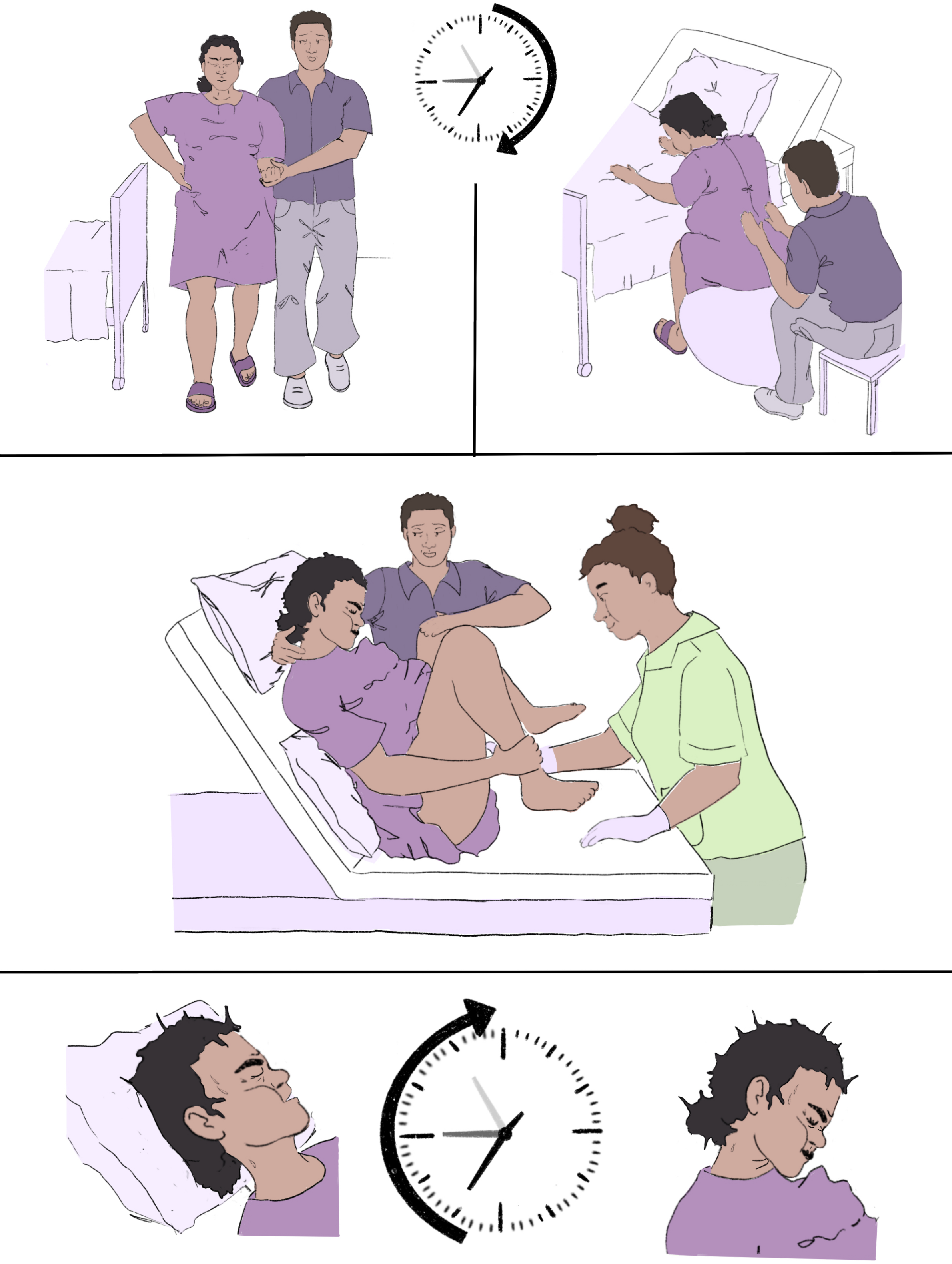

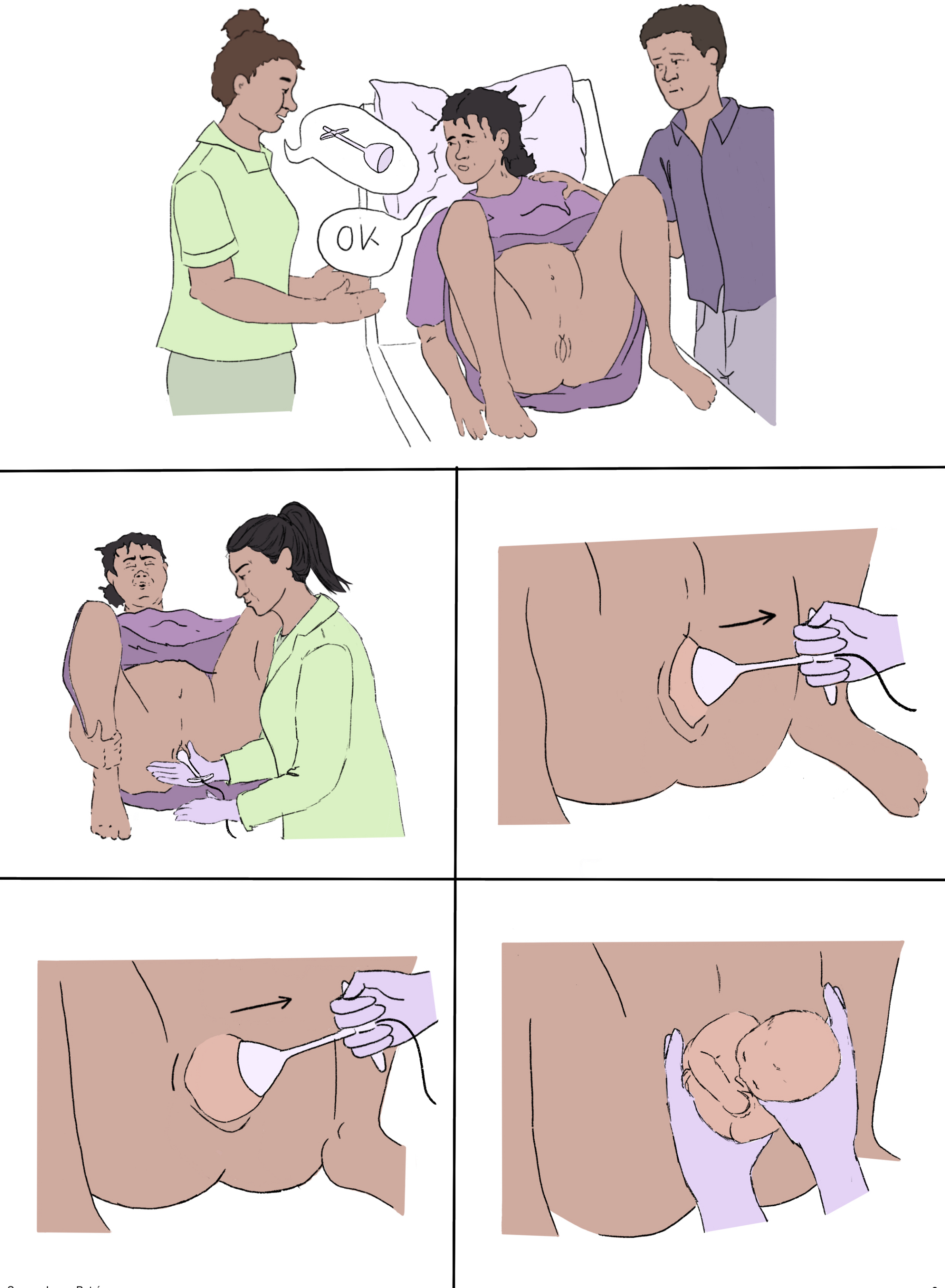
OK

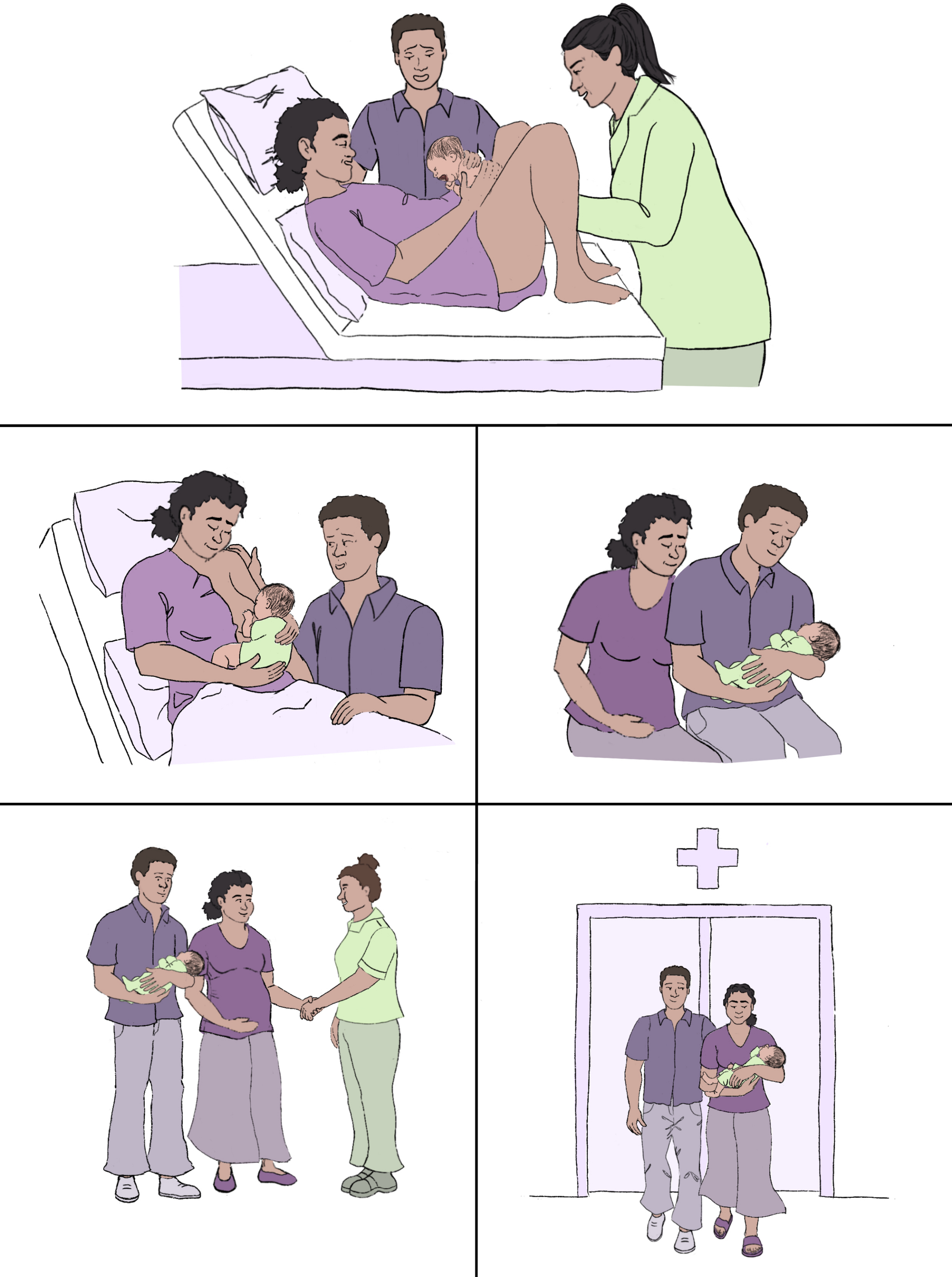

Comics depicting forcep use

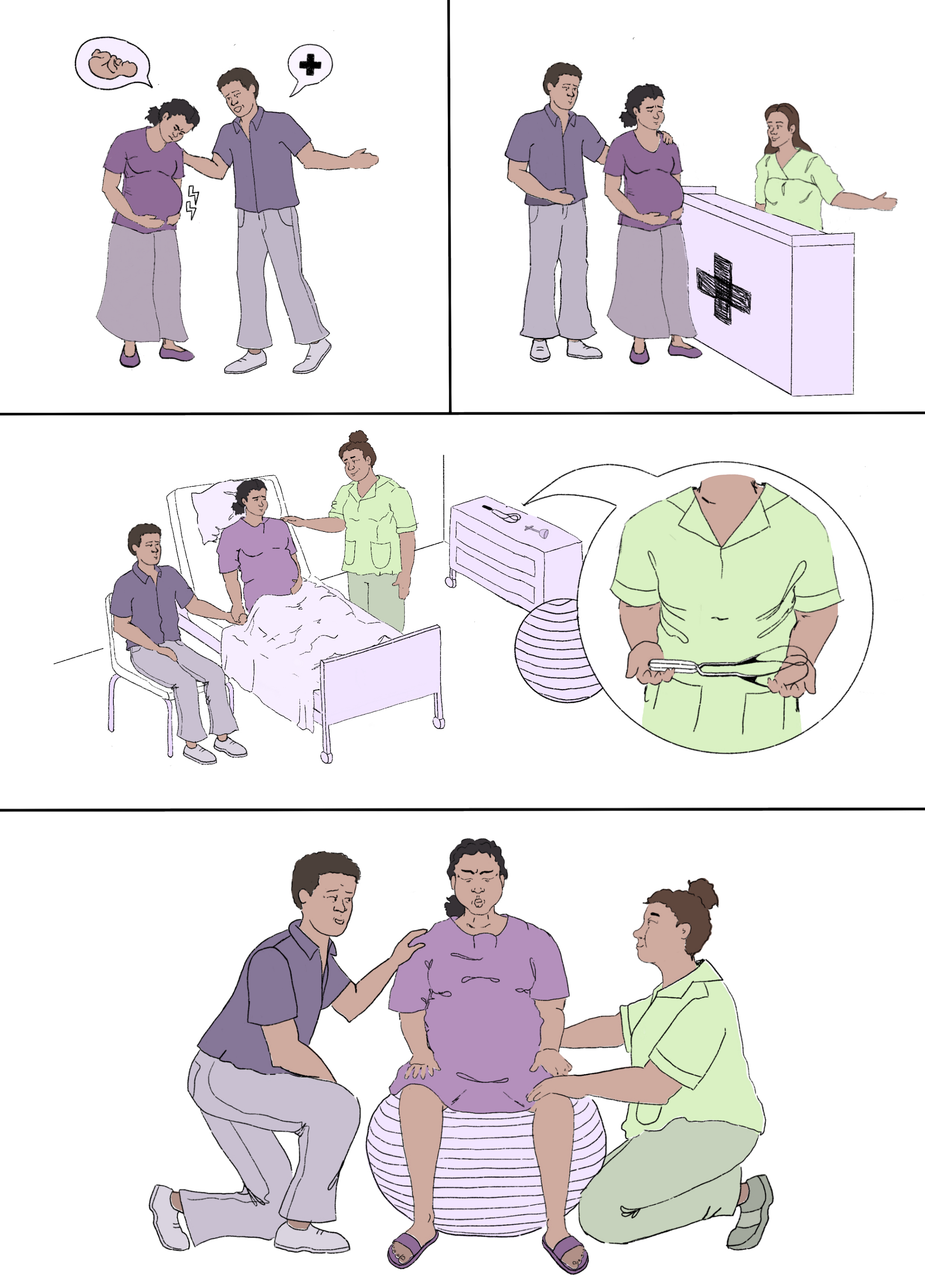

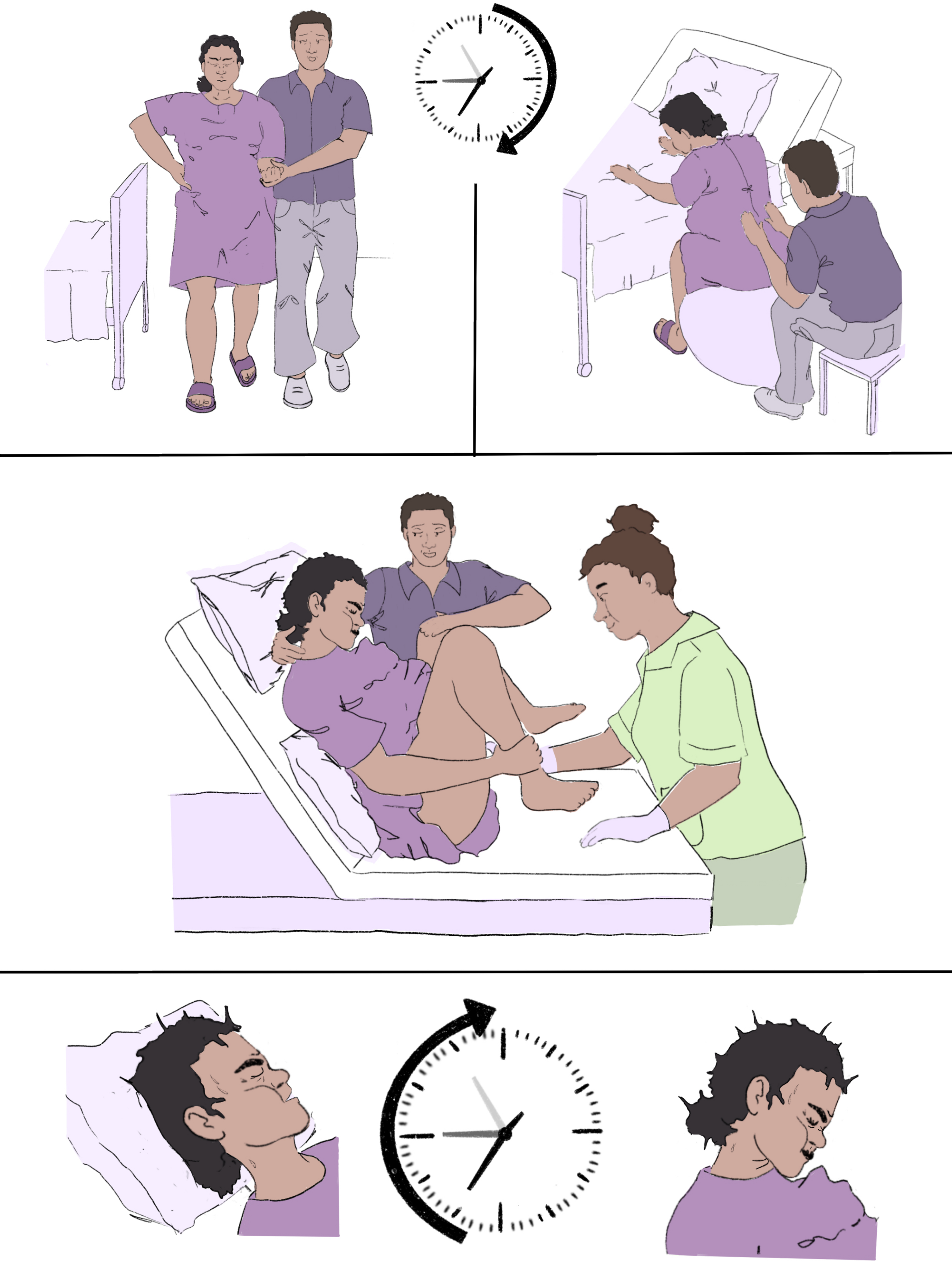

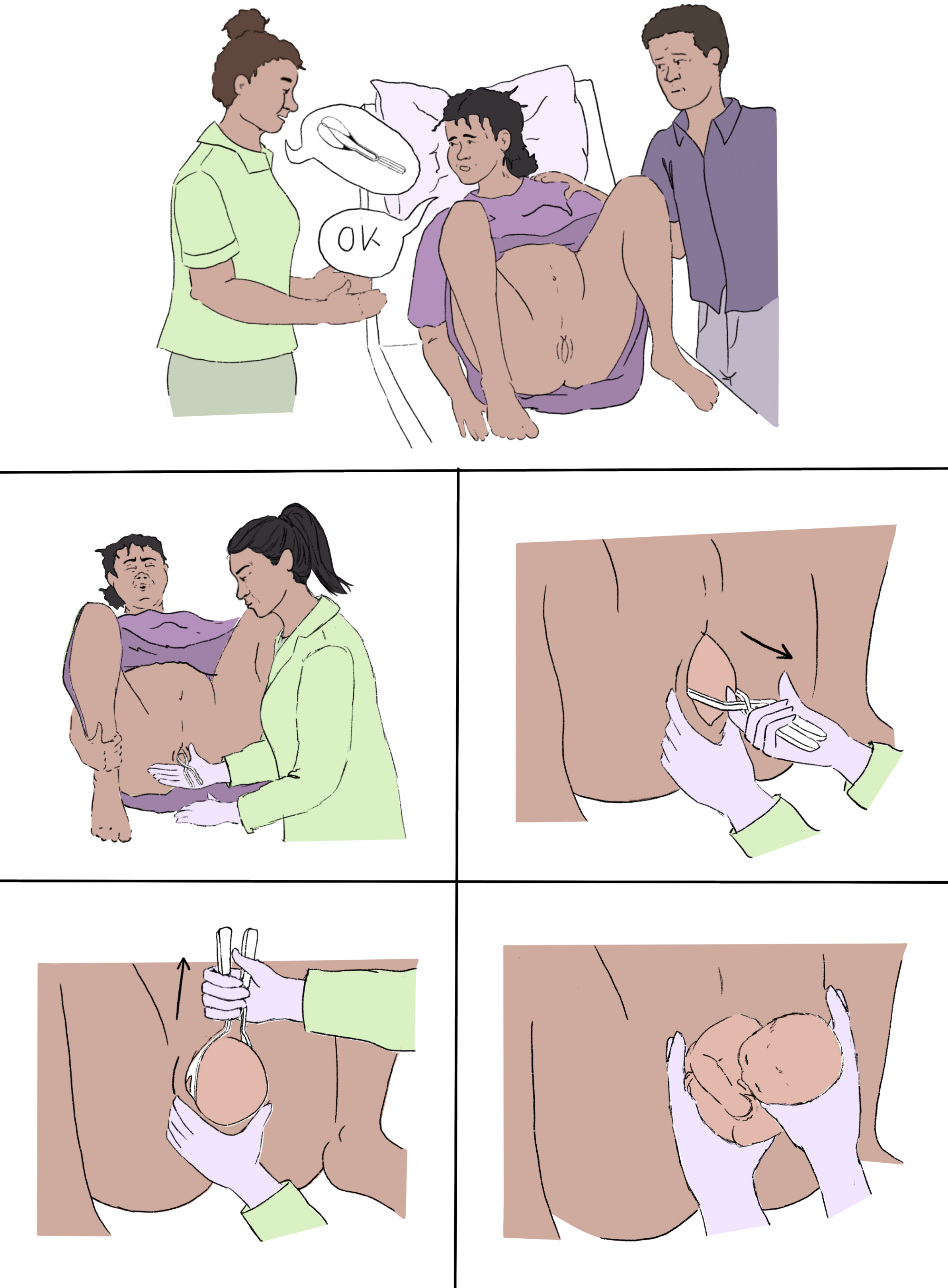
OK

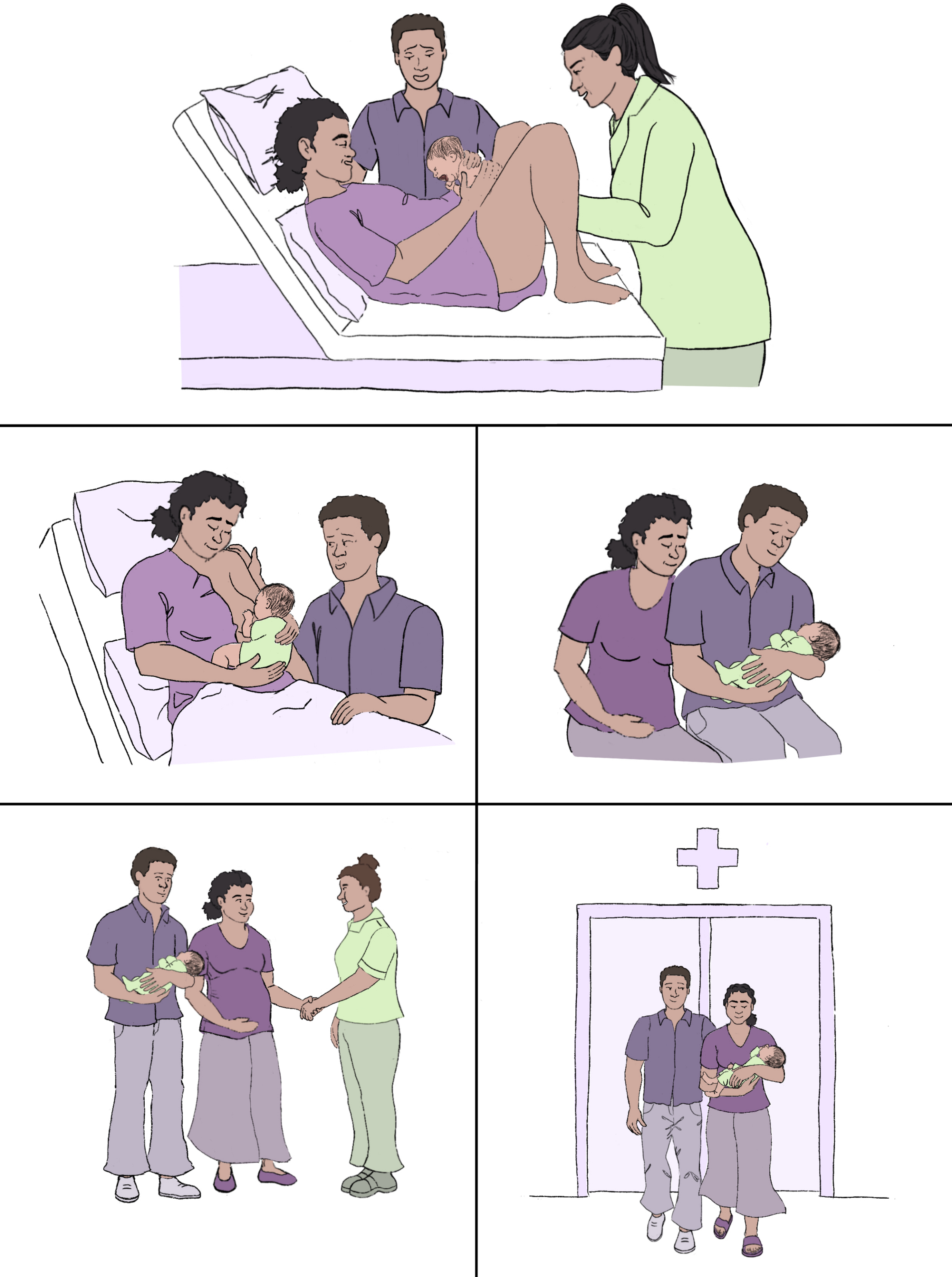

Supplement: Multimedia Appendix 3 [file resprot_v14i1e69808_app3.pdf]
