# Supplementary material for: Optimizing Availability and Appropriate Use of Assisted Vaginal Birth: Protocol for Generic Formative Research of an Implementation Preparation
Source: JMIR Res Protoc. 2025 Sep 8;14:e69808. doi: 10.2196/69808 (PMC12455161; doi:10.2196/69808)
Supplement: Multimedia Appendix 4 [file resprot_v14i1e69808_app4.docx]

**Frequently Asked Questions About Assisted Vaginal Birth**

Adapted from Royal College of Obstetricians & Gynaecologists: <https://www.rcog.org.uk/media/2p4fh2kd/pi-vaginal-birth-final-28042020.pdf>

**1. What is an assisted vaginal birth?**

An assisted vaginal birth is when a doctor or midwife uses specially designed instruments to help deliver your baby. These instruments can include:

- **Forceps:** smooth and curved metal instruments that look like large tongs. They are gently inserted into the vagina and positioned around the baby's head to help guide the baby out. During contractions, you will push while the healthcare professional pulls with the forceps to help deliver your baby.
- **Ventouse (vacuum extraction):** a cup that is attached to the baby’s head. The cup is connected to a vacuum pump that helps pull the baby out. During contractions, you will be asked to push while the healthcare professional pulls to assist the birth.

The choice of the instrument depends on various factors such as the baby’s position and the effectiveness of any epidural you may have had.

**2. Why might an assisted vaginal birth be needed?**

There are several reasons why a doctor or midwife might recommend an assisted vaginal birth:

- Labor is not progressing adequately.
- The baby shows signs of distress.
- You are unable to, or have been advised not to, push during birth.
- Certain medical conditions require a faster delivery.

**3. What are my alternatives to assisted vaginal birth?**

Forceps or a ventouse will only be recommended if they’re the safest option. Your healthcare provider will discuss the reasons, the procedure, and your options at the time. If you choose not to have an assisted vaginal birth, you can wait for a natural birth or opt for an emergency caesarean. Remember, a caesarean during the late stage of labour is more complex and riskier than a planned one. Decision-making in labour can be difficult, which is why it is important to explore any concerns you may have with your healthcare professional before you go into labour. If you’re certain about avoiding an assisted vaginal birth, consider discussing a planned caesarean during pregnancy.

**4. What happens during an assisted vaginal birth?**

- With your consent, the doctor or midwife will check your abdomen and do an internal exam to make sure assisted vaginal birth is safe for you and your baby.
- You may get pain relief
- You might need a small cut on your vagina (episiotomy) to make your vagina opening larger and allow your baby to be born.
- A newborn care specialist may be there to help with your baby if needed.
- If your baby is well, you can have immediate skin to skin contact and/or delayed cord clamping right after your baby is born.

**5. What makes an assisted vaginal birth less likely to be successful?**

Assisted vaginal birth is less likely to be successful if:

- You are overweight with a body mass index (BMI) over 30
- You are less than 161cm in height
- Your baby is estimated to be more than 4kg in weight
- Your baby is lying with its back to your back at the end of your labour
- Your baby’s head is not low down in the birth canal at the end of your labour

**6. What will an assisted vaginal birth mean for me?**

- Longer hospital stays than originally expected.
- Increased bleeding immediately after birth, which is expected after assisted vaginal birth.
- Higher chance of needing an episiotomy or having vaginal tears.
- Most women have some discomfort or pain after the procedure.
- Potential urinary issues and need for catheterisation.

**7. What will an assisted vaginal birth mean for my baby?**

Most babies are well and do not have long-term problems. Marks from the instruments usually disappear within 24-48 hours. Rare risks include jaundice, bleeding in the brain, and serious trauma.

**8. How will I feel after leaving the hospital?**

Most women recover well. Pain and discomfort are common but manageable with regular pain relief. You can begin to have sex again when you feel that it’s the right time for you,

Birth can be distressing, and some women may risk developing PTSD. If you experience anxiety, low mood, or need support after an assisted vaginal birth, talk to your healthcare professional.

**9. Will I need an assisted vaginal birth next time?**

Having an assisted vaginal birth does not mean you will need one in your next pregnancy. Most women who have an assisted vaginal birth have a vaginal birth next time round without needing assistance.
