# Supplementary material for: Optimizing Availability and Appropriate Use of Assisted Vaginal Birth: Protocol for Generic Formative Research of an Implementation Preparation
Source: JMIR Res Protoc. 2025 Sep 8;14:e69808. doi: 10.2196/69808 (PMC12455161; doi:10.2196/69808)
Supplement: Multimedia Appendix 5 [file resprot_v14i1e69808_app5.docx]

# Plain Language Statement For Women and Women’s Organisations

***Optimising availability and appropriate use of assisted vaginal birth: a generic formative research protocol for implementation preparation***

### Introduction

Thank you for your interest in participating in this research project. The following pages will provide you with information about the project, so that you can decide if you would like to take part in this research.

Please take the time to read this information carefully. You may ask questions about anything you don’t understand or want to know more about.

Your participation is voluntary. If you don’t wish to take part, you don’t have to. If you begin participating, you can also stop at any time.

### What is this research about?

Assisted vaginal birth is a procedure where doctors or midwives use special tools like forceps or vacuum extractors to help women give birth. Assisted vaginal birth is recommended when there are difficulties during birth, like if the woman is too tired to push. While assisted vaginal births can help women recover faster and have fewer problems after birth compared to caesarean section, there are some risks. These include ears, pelvic problems, and infections. Babies born through assisted vaginal birth may also have small cuts or bruises. Even though these risks can happen, they are rare. We can minimise these risks by choosing the right people for assisted vaginal birth and having skilled doctors or nurses perform the procedure gently. When done right, assisted vaginal birth can lead to a quicker recovery, shorter hospital stays, lower risks of bleeding and infection, and it increases the chance of having a natural birth in future pregnancies.

Assisted vaginal birth has clear benefits, but it has been used less often lately. At the same time, the number of cesarean births has been going up all around the world. In 2018, more than 21% of women worldwide had a caesarean, and this number is expected to keep going up until 2030. Making assisted vaginal birth more available and better quality could help reduce the chances of problems for both moms and babies without having to do a caesarean. This could be especially helpful in places with fewer resources, like small clinics or places where it's hard to get to a hospital quickly. Sometimes, it's not safe or easy to do a caesarean, or it takes too long to get to a bigger hospital for one.

But even though there are possibilities for improving assisted vaginal birth, there are still challenges and things we don't know enough about. For example, we're not sure how to make sure assisted vaginal birth happens well, fairly, and for lots of people in places with fewer resources. This study aims to talk to women like you to understand what you think about assisted vaginal birth. We want to know how you make decisions about childbirth and how comfortable you are with getting information about assisted vaginal birth. By talking to you, we hope to figure out the best ways to give women information about assisted vaginal birth and make sure it is done in a way that works for everyone.

This research is funded by [insert funder details].

### What will I be asked to do?

Should you agree to participate, you will be asked to join one interview or focus group discussion. The interview will cover questions about your personal background, your birth experiences, and your views on assisted vaginal birth, including how information about it is shared. The focus group discussion will specifically address your opinions and perceptions of assisted vaginal birth and the related information sharing. If you agree to participate, you will be asked to sign a consent form or record verbal consent stating that you agree to participate and to take part in an interview. The interview or discussion. will be conducted at a place agreed upon between you and the researcher, and, most importantly, it will be comfortable for you. All the conversations taking place in the interview will be audio recorded, and the researcher may record written notes as well. The interview will take around 45 to 60 minutes, while the focus group discussion will last approximately between 60 to 90 minutes*.* You will be reimbursed with [insert details].

### What are the possible benefits?

There are no direct benefits to participating in this interview. This interview will assist us in learning more about the views, preferences, experiences, and priorities on assisted vaginal birth. This information can help us better understand how to formulate effective and efficient guidelines as well as interventions in optimising assisted vaginal birth use.

### What are the possible risks?

The discussion around childbirth can be unpleasant for some people, may induce anxiety and fears among women with the first pregnancy, as well as trigger painful memories for women with previous childbirth experience which may make you feel uncomfortable. Therefore, you are free to decide whether you want to participate in this research or not. If you decide to participate in this study, you can tell the researcher if you experience any discomfort during the interview or discussion, you can also decide if you still would like to proceed, pause, or stop the conversation. Please also be assured that you do not need to answer any questions that you feel uncomfortable answering. If you are experiencing any emotional distress during the interview, our research team can support referral to the appropriate health service or contact your family members under your permission. If you still feel emotional difficulties after the interview or discussion finishes, we encourage you to seek assistance by contacting the mental health counselling service where you live.

During focus group discussions, you will engage in conversations with other participants about childbirth-related topics. These discussions will be facilitated by a researcher who will guide the conversation and encourage everyone to share their thoughts and experiences openly. However, this means that there is a risk to confidentiality through the due to the possibility of other study participants disclosing information. We will try to minimise this by emphasising the importance to respect the privacy of all participants by keeping the information shared in the discussion confidential. We will request participants to respect the privacy of other participants by keeping the information shared in the discussion to themselves.

Additionally, to protect your privacy, we will not mention any of your personal details, such as your name and contact details, in any published reports. However, we may use a chunk of sentences from your speech in the interview called a “quote” on the published reports, with a made-up identification. Even with this made-up identification, there is a risk that some people might be able to identify you as there will only be a small number of women participating in this study.

### What will happen to information about me?

Any information collected about you will be protected and will be treated as confidential. Your information can only be shared with other parties under your permission or when required by law. Your data will be de-identified before analysis takes place. The original audio-recorded data will be deleted after the transcription to avoid identification. Your contact information and your data will not be able to be connected by other people outside of the research team and will be kept securely in locked cupboards and electronically at a secured institution-owned online drive and will be protected by a password. All data will be securely kept in the [insert institution name] for [insert specific retention period based on local regulations/requirements] from the study completion, and will be deleted or destroyed after this time. The responsible researcher will be the one managing and protecting this data, and no access will be given to the non-processed data to the people outside of the research team. However, it is possible that the de-identified data (data with your personal information removed) may be used in the future for different purposes. These could include additional studies to look for new patterns, compare them with other studies, create case studies, or use them in educational materials for teaching or meetings.

### Do I have to take part?

No. Participation in this study is completely voluntary. You can decide if you would like to answer or not to answer some questions in the interview. In addition, you are also free to withdraw or quit the study at any time. If you choose to withdraw your participation in this study, you can withdraw any unprocessed data within 2 weeks after you have given the data to the researcher, however, after this and once the data is processed, we would like to use the information you have provided until your withdrawal. Your relationship with any people or organisations in this study will not be impacted if you quit the study. If you wish, you may also discuss your decision with your partner or family member before proceeding.

### Will I hear about the results of this project?

The results of this study will be published in academic journals, and online media, as well as shared with organisations involved in this study. The study team will share the results of the study with you, you can provide an email address or WhatsApp contact if you do not have an email, and the researcher will send a study report to you through the contact provided.

### Where can I get further information?

If you would like more information about the project, please contact the researcher; [insert researcher contact]

### Who can I contact if I have any concerns about the project?

This research project has been approved by [insert ethics approval details]. If you have any concerns or complaints about the conduct of this research project, which you do not wish to discuss with the research team, you should contact [insert ethics committee detail]. In any correspondence, please provide the name of the research team or the name or ethics ID number of the research project.

# Plain Language Statement For Family and Community Members

***Optimising availability and appropriate use of assisted vaginal birth: a generic formative research protocol for implementation preparation***

### Introduction

Thank you for your interest in participating in this research project. The following pages will provide you with information about the project so that you can decide if you would like to take part in this research.

Please take the time to read this information carefully. You may ask questions about anything you don’t understand or want to know more about.

Your participation is voluntary. If you don’t wish to take part, you don’t have to. If you begin participating, you can also stop at any time.

### What is this research about?

Assisted vaginal birth is a procedure where doctors or midwives use special tools like forceps or vacuum extractors to help women give birth. Assisted vaginal birth is recommended when there are difficulties during birth, like if the woman is too tired to push. While assisted vaginal births can help women recover faster and have fewer problems after birth compared to caesarean section, there are some risks. These include ears, pelvic problems, and infections. Babies born through assisted vaginal birth may also have small cuts or bruises. Even though these risks can happen, they are rare. We can minimise these risks by choosing the right people for assisted vaginal birth and having skilled doctors or nurses perform the procedure gently. When done right, assisted vaginal birth can lead to a quicker recovery, shorter hospital stays, lower risks of bleeding and infection, and it increases the chance of having a natural birth in future pregnancies.

Assisted vaginal birth has clear benefits, but it has been used less often lately. At the same time, the number of cesarean births has been going up all around the world. In 2018, more than 21% of women worldwide had a caesarean, and this number is expected to keep going up until 2030. Making assisted vaginal birth more available and better quality could help reduce the chances of problems for both moms and babies without having to do a caesarean. This could be especially helpful in places with fewer resources, like small clinics or places where it's hard to get to a hospital quickly. Sometimes, it's not safe or easy to do a caesarean, or it takes too long to get to a bigger hospital for one.

But even though there are possibilities for improving assisted vaginal birth, there are still challenges and things we don't know enough about. For example, we're not sure how to make sure assisted vaginal birth happens well, fairly, and for lots of people in places with fewer resources. This study aims to talk to healthcare providers like you to understand what you think about assisted vaginal birth. We want to know how acceptable you find different ways of improving assisted vaginal birth and what strategies might work best to make those improvements happen. By talking to you, we hope to figure out the best ways to make sure more women can benefit from assisted vaginal birth and that it's done in a way that works for everyone.

This research is funded by [insert funder details].

### What will I be asked to do?

Should you agree to participate, you will be asked to join one focus group discussion. The interview will include questions about yourself, your views, and perceptions on assisted vaginal birth and information sharing associated with it. If you agree to participate, you will be asked to sign a consent form or record verbal consent stating that you agree to participate and to take part in an interview. The interview or discussion. will be conducted at a place agreed upon between you and the researcher, and, most importantly, it will be comfortable for you. All the conversations taking place in the discussion will be audio recorded, and the researcher may record written notes as well. The focus group discussion will last approximately between 60 to 90 minutes. You will be reimbursed with [insert details].

### What are the possible benefits?

There are no direct benefits to participating in this interview. This interview will assist us in learning more about the views, preferences, experiences, and priorities on assisted vaginal birth. This information can help us better understand how to formulate effective and efficient guidelines as well as interventions in optimising assisted vaginal birth use.

### What are the possible risks?

The discussion around childbirth can be unpleasant for some people, may induce anxiety and fears, as well as trigger painful memories for women with previous childbirth experience which may make you feel uncomfortable. Therefore, you are free to decide whether you want to participate in this research or not. If you decide to participate in this study, you can tell the researcher if you experience any discomfort during the discussion, you can also decide if you still would like to proceed, pause, or stop the conversation. Please also be assured that you do not need to answer any questions that you feel uncomfortable answering. If you are experiencing any emotional distress during the interview, our research team can support referral to the appropriate health service or contact your family members under your permission. If you still feel emotional difficulties after the interview or discussion finishes, we encourage you to seek assistance by contacting the mental health counselling service where you live.

During focus group discussions, you will engage in conversations with other participants about childbirth-related topics. These discussions will be facilitated by a researcher who will guide the conversation and encourage everyone to share their thoughts and experiences openly. However, this means that there is a risk to confidentiality through the due to the possibility of other study participants disclosing information. We will try to minimise this by emphasising the importance to respect the privacy of all participants by keeping the information shared in the discussion confidential. We will request participants to respect the privacy of other participants by keeping the information shared in the discussion to themselves.

Additionally, to protect your privacy, we will not mention any of your personal details, such as your name and contact details, in any published reports. However, we may use a chunk of sentences from your speech in the interview called a “quote” on the published reports, with a made-up identification. Even with this made-up identification, there is a risk that some people might be able to identify you as there will only be a small number of women participating in this study.

### What will happen to information about me?

Any information collected about you will be protected and will be treated as confidential. Your information can only be shared with other parties under your permission or when required by law. Your data will be de-identified before analysis takes place. The original audio-recorded data will be deleted after the transcription to avoid identification. Your contact information and your data will not be able to be connected by other people outside of the research team and will be kept securely in locked cupboards and electronically at a secured institution-owned online drive and will be protected by a password. All data will be securely kept in the [insert institution name] for [insert specific retention period based on local regulations/requirements] from the study completion, and will be deleted or destroyed after this time. The responsible researcher will be the one managing and protecting this data and there will be no access given to the non-processed data to the people outside of the research team. However, it is possible that the de-identified data (data with your personal information removed) may be used in the future for different purposes. These could include additional studies to look for new patterns, compare them with other studies, create case studies, or use them in educational materials for teaching or meetings.

### Do I have to take part?

No. Participation in this study is completely voluntary. You can decide if you would like to answer or not answer some questions in the interview. In addition, you are also free to withdraw or quit the study at any time. If you choose to withdraw your participation in this study, you can withdraw any unprocessed data within 2 weeks after you have given the data to the researcher, however, after this and once the data is processed, we would like to use the information you have provided until your withdrawal. Your relationship with any people or organisations in this study will not be impacted if you quit the study. If you wish, you may also discuss your decision with your partner or family member before proceeding.

### Will I hear about the results of this project?

The results of this study will be published in academic journals, and online media, as well as shared with organisations involved in this study. The study team will share the results of the study with you, you can provide an email address or WhatsApp contact if you do not have an email, and the researcher will send a study report to you through the contact provided.

### Where can I get further information?

If you would like more information about the project, please contact the researcher; [insert researcher contact]

### Who can I contact if I have any concerns about the project?

This research project has been approved by [insert ethics approval details]. If you have any concerns or complaints about the conduct of this research project, which you do not wish to discuss with the research team, you should contact [insert ethics committee detail]. In any correspondence, please provide the name of the research team or the name or ethics ID number of the research project.

# Plain Language Statement For Providers and Administrators

***Optimising availability and appropriate use of assisted vaginal birth: a generic formative research protocol for implementation preparation***

### Introduction

Thank you for your interest in participating in this research project. The following pages will provide you with information about the project so that you can decide if you would like to take part in this research.

Please take the time to read this information carefully. You may ask questions about anything you don’t understand or want to know more about.

Your participation is voluntary. If you don’t wish to take part, you don’t have to. If you begin participating, you can also stop at any time.

### What is this research about?

Assisted vaginal birth is a procedure where doctors or midwives use special tools like forceps or vacuum extractors to help women give birth. Assisted vaginal birth is recommended when there are difficulties during birth, like if the woman is too tired to push. While assisted vaginal births can help women recover faster and have fewer problems after birth compared to caesarean section, there are some risks. These include ears, pelvic problems, and infections. Babies born through assisted vaginal birth may also have small cuts or bruises. Even though these risks can happen, they are rare. We can minimise these risks by choosing the right people for assisted vaginal birth and having skilled doctors or nurses perform the procedure gently. When done right, assisted vaginal birth can lead to a quicker recovery, shorter hospital stays, lower risks of bleeding and infection, and it increases the chance of having a natural birth in future pregnancies.

Assisted vaginal birth has clear benefits, but it has been used less often lately. At the same time, the number of cesarean births has been going up all around the world. In 2018, more than 21% of women worldwide had a caesarean, and this number is expected to keep going up until 2030. Making assisted vaginal birth more available and better quality could help reduce the chances of problems for both moms and babies without having to do a caesarean. This could be especially helpful in places with fewer resources, like small clinics or places where it's hard to get to a hospital quickly. Sometimes, it's not safe or easy to do a caesarean, or it takes too long to get to a bigger hospital for one.

But even though there are possibilities for improving assisted vaginal birth, there are still challenges and things we don't know enough about. For example, we're not sure how to make sure assisted vaginal birth happens well, fairly, and for lots of people in places with fewer resources. This study aims to talk to healthcare providers like you to understand what you think about assisted vaginal birth. We want to know how acceptable you find different ways of improving assisted vaginal birth and what strategies might work best to make those improvements happen. By talking to you, we hope to figure out the best ways to make sure more women can benefit from assisted vaginal birth and that it's done in a way that works for everyone.

This research is funded by [insert funder details].

### What will I be asked to do?

Should you agree to participate, you will be asked to join one to one interview. The interview will include questions about yourself, your views, and perceptions on assisted vaginal birth and potential interventions to optimise its use. If you agree to participate, you will be asked to sign a consent form or record verbal consent stating that you agree to participate and to take part in an interview. The interview or discussion. will be conducted at a place agreed between you and the researcher, and importantly, will be comfortable for you. All the conversations taking place in the interview will be audio recorded and the researcher may record written notes as well. The interview will take around 45 minutes to one hour*.* You will be reimbursed with [insert details].

### What are the possible benefits?

There are no direct benefits to participating in this study. This study will assist us in learning more about the views, preferences, experiences, and priorities of assisted vaginal birth. This information can help us better understand how to formulate effective and efficient guidelines as well as interventions in optimising assisted vaginal birth use.

### What are the possible risks?

The discussion around childbirth can be unpleasant for some people and may trigger painful memories which may make you feel uncomfortable. Therefore, you are free to decide whether you want to participate in this research or not. If you decide to participate in this study, you can tell the researcher if you experience any discomfort during the interview or discussion, you can also decide if you still would like to proceed, pause, or stop the conversation. Please also be assured that you do not need to answer any questions that you feel uncomfortable answering. If you are experiencing any emotional distress during the interview, our research team can support referral to the appropriate health service or contact your family members under your permission. If you still feel emotional difficulties after the interview finishes, we encourage you to seek assistance by contacting the mental health counselling service where you live.

Additionally, to protect your privacy, we will not mention any of your personal details, such as your name and contact details, in any published reports. However, we may use a chunk of sentences from your speech in the interview called a “quote” on the published reports, with a made-up identification. Even with this made-up identification, there is a risk that some people might be able to identify you as there will only be a small number of women participating in this study.

### What will happen to information about me?

Any information collected about you will be protected and will be treated as confidential. Your information can only be shared with other parties under your permission or when required by law. Your data will be de-identified before analysis takes place. The original audio-recorded data will be deleted after the transcription to avoid identification. Your contact information and your data will not be able to be connected by other people outside of the research team and will be kept securely in locked cupboards and electronically at a secured institution-owned online drive and will be protected by a password. All data will be securely kept in the [insert institution name] for [insert specific retention period based on local regulations/requirements] from the study completion, and will be deleted or destroyed after this time. The responsible researcher will be the one managing and protecting this data and there will be no access given to the non-processed data to the people outside of the research team. However, it is possible that the de-identified data (data with your personal information removed) may be used in the future for different purposes. These could include additional studies to look for new patterns, compare them with other studies, create case studies, or use them in educational materials for teaching or meetings.

### Do I have to take part?

No. Participation in this study is completely voluntary. You can decide if you would like to answer or not answer some questions in the interview. In addition, you are also free to withdraw or quit the study at any time. If you choose to withdraw your participation in this study, you can withdraw any unprocessed data within 2 weeks after you have given the data to the researcher, however, after this and once the data is processed, we would like to use the information you have provided until your withdrawal. Your relationship with any people or organisations in this study will not be impacted if you quit the study. If you wish, you may also discuss your decision with your partner or family member before proceeding.

### Will I hear about the results of this project?

The results of this study will be published in academic journals, and online media, as well as shared with organisations involved in this study. The study team will share the results of the study with you, you can provide an email address or WhatsApp contact if you do not have an email, and the researcher will send a study report to you through the contact provided.

### Where can I get further information?

If you would like more information about the project, please contact the researcher; [insert researcher contact]

### Who can I contact if I have any concerns about the project?

This research project has been approved by [insert ethics approval details]. If you have any concerns or complaints about the conduct of this research project, which you do not wish to discuss with the research team, you should contact [insert ethics committee detail]. In any correspondence, please provide the name of the research team or the name or ethics ID number of the research project.

# Plain Language Statement For Policymakers

***Optimising availability and appropriate use of assisted vaginal birth: a generic formative research protocol for implementation preparation***

### Introduction

Thank you for your interest in participating in this research project. The following pages will provide you with information about the project so that you can decide if you would like to take part in this research.

Please take the time to read this information carefully. You may ask questions about anything you don’t understand or want to know more about.

Your participation is voluntary. If you don’t wish to take part, you don’t have to. If you begin participating, you can also stop at any time.

### What is this research about?

Assisted vaginal birth is a procedure where doctors or midwives use special tools like forceps or vacuum extractors to help women give birth. Assisted vaginal birth is recommended when there are difficulties during birth, like if the woman is too tired to push. While assisted vaginal births can help women recover faster and have fewer problems after birth compared to caesarean section, there are some risks. These include ears, pelvic problems, and infections. Babies born through assisted vaginal birth may also have small cuts or bruises. Even though these risks can happen, they are rare. We can minimise these risks by choosing the right people for assisted vaginal birth and having skilled doctors or nurses perform the procedure gently. When done right, assisted vaginal birth can lead to a quicker recovery, shorter hospital stays, lower risks of bleeding and infection, and it increases the chance of having a natural birth in future pregnancies.

Assisted vaginal birth has clear benefits, but it has been used less often lately. At the same time, the number of cesarean births has been going up all around the world. In 2018, more than 21% of women worldwide had a caesarean, and this number is expected to keep going up until 2030. Making assisted vaginal birth more available and better quality could help reduce the chances of problems for both moms and babies without having to do a caesarean. This could be especially helpful in places with fewer resources, like small clinics or places where it's hard to get to a hospital quickly. Sometimes, it's not safe or easy to do a caesarean, or it takes too long to get to a bigger hospital for one.

But even though there are possibilities for improving assisted vaginal birth, there are still challenges and things we don't know enough about. For example, we're not sure how to make sure assisted vaginal birth happens well, fairly, and for lots of people in places with fewer resources. This study aims to talk to policymakers like you to understand what you think about assisted vaginal birth. We want to know how acceptable you find different ways of improving assisted vaginal birth and what strategies might work best to keep those improvements going. By talking to you, we hope to figure out the best ways to make sure more women can benefit from assisted vaginal birth and that it's done in a way that works for everyone.

This research is funded by [insert funder details].

### What will I be asked to do?

Should you agree to participate, you will be asked to join one interview. The interview will include questions about yourself, your views, and perceptions on assisted vaginal birth and potential interventions to optimise its use. If you agree to participate, you will be asked to sign a consent form or record verbal consent stating that you agree to participate and to take part in an interview. The interview or discussion. will be conducted at a place agreed between you and the researcher, and importantly, will be comfortable for you. All the conversations taking place in the interview will be audio recorded and the researcher may record written notes as well. The interview will take around 45 minutes to one hour*.* You will be reimbursed with [insert details].

### What are the possible benefits?

There are no direct benefits to participating in this interview. The interview will assist us in learning more about the views, preferences, experiences, and priorities on assisted vaginal birth. This information can help us better understand how to formulate effective and efficient guidelines as well as interventions in optimising assisted vaginal birth use.

### What are the possible risks?

The discussion around childbirth can be unpleasant for some people and may trigger painful memories which may make you feel uncomfortable. Therefore, you are free to decide whether you want to participate in this research or not. If you decide to participate in this study, you can tell the researcher if you experience any discomfort during the interview or discussion, you can also decide if you still would like to proceed, pause, or stop the conversation. Please also be assured that you do not need to answer any questions that you feel uncomfortable answering. If you are experiencing any emotional distress during the interview, our research team can support referral to the appropriate health service or contact your family members under your permission. If you still feel emotional difficulties after the interview finishes, we encourage you to seek assistance by contacting the mental health counselling service where you live.

Additionally, to protect your privacy, we will not mention any of your personal details, such as your name and contact details, in any published reports. However, we may use a chunk of sentences from your speech in the interview called a “quote” on the published reports, with a made-up identification. Even with this made-up identification, there is a risk that some people might be able to identify you as there will only be a small number of women participating in this study.

### What will happen to information about me?

Any information collected about you will be protected and will be treated as confidential. Your information can only be shared with other parties under your permission or when required by law. Your data will be de-identified before analysis takes place. The original audio-recorded data will be deleted after the transcription to avoid identification. Your contact information and your data will not be able to be connected by other people outside of the research team and will be kept securely in locked cupboards and electronically at a secured institution-owned online drive and will be protected by a password. All data will be securely kept in the [insert institution name] for [insert specific retention period based on local regulations/requirements] from the study completion, and will be deleted or destroyed after this time. The responsible researcher will be the one managing and protecting this data and there will be no access given to the non-processed data to the people outside of the research team. However, it is possible that the de-identified data (data with your personal information removed) may be used in the future for different purposes. These could include additional studies to look for new patterns, compare them with other studies, create case studies, or use them in educational materials for teaching or meetings.

### Do I have to take part?

No. Participation in this study is completely voluntary. You can decide if you would like to answer or not answer some questions in the interview. In addition, you are also free to withdraw or quit the study at any time. If you choose to withdraw your participation in this study, you can withdraw any unprocessed data within 2 weeks after you have given the data to the researcher, however, after this and once the data is processed, we would like to use the information you have provided until your withdrawal. Your relationship with any people or organisations in this study will not be impacted if you quit the study. If you wish, you may also discuss your decision with your partner or family member before proceeding.

### Will I hear about the results of this project?

The results of this study will be published in academic journals, and online media, as well as shared with organisations involved in this study. The study team will share the results of the study with you, you can provide an email address or WhatsApp contact if you do not have an email, and the researcher will send a study report to you through the contact provided.

### Where can I get further information?

If you would like more information about the project, please contact the researcher; [insert researcher contact]

### Who can I contact if I have any concerns about the project?

This research project has been approved by [insert ethics approval details]. If you have any concerns or complaints about the conduct of this research project, which you do not wish to discuss with the research team, you should contact [insert ethics committee detail]. In any correspondence, please provide the name of the research team or the name or ethics ID number of the research project.
