# Supplementary material for: Optimizing Availability and Appropriate Use of Assisted Vaginal Birth: Protocol for Generic Formative Research of an Implementation Preparation
Source: JMIR Res Protoc. 2025 Sep 8;14:e69808. doi: 10.2196/69808 (PMC12455161; doi:10.2196/69808)
Supplement: Multimedia Appendix 6 [file resprot_v14i1e69808_app6.docx]

# Consent Form

***Optimising availability and appropriate use of assisted vaginal birth: a generic formative research protocol for implementation preparation.***

| **Name of Participant:** |  |
| --- | --- |

1. I agree to participate in this project. I have been given a clear explanation of the details and a written summary to keep.
2. I understand that this research aims to learn about my views and preferences on assisted vaginal birth, including how I feel about different ways to improve its use.
3. I know that my participation is only for research purposes.
4. I have been told about the possible effects of participating, and I am satisfied with the explanations provided.
5. I will be asked to join a one-on-one interview or focus group discussion.
6. The interview or focus group discussion will take about 45 to 90 minutes and can be done face-to-face or online at a time that works for both me and the researcher.
7. I understand that my interview or discussion will be recorded and transcribed for analysis.
8. I know my participation is voluntary, and I can withdraw at any time without giving a reason or facing any consequences. I can also request to remove any unprocessed data I provided within two weeks after the interview or discussion.
9. I understand that my data will be stored at [insert institution name] and destroyed after [insert specific retention period based on local regulations/requirements].
10. I have been informed that my information will be kept confidential (subject to legal requirements), protected by a password, and only accessible to the named researchers.
11. I understand that de-identified data (data with my personal information removed) may be used in the future for other purposes, such as finding new patterns, comparing with other studies, creating case studies, or teaching and educational materials.
12. I understand that I will receive [insert reimbursement details for participants] as a thank you for my participation.
13. I have been told that this project is funded by [insert funder details].
14. I know that after I sign and return this consent form, the researcher will keep it.
15. By signing this form, I confirm that I have read and understood the information provided. If I cannot read or write, a member of the research team will read the information to me and ask for my verbal consent, which will be recorded as proof of my agreement to participate.

| **Participant Signature:** |  | **Date:** |  |
| --- | --- | --- | --- |
