# Supplementary material for: Optimizing Availability and Appropriate Use of Assisted Vaginal Birth: Protocol for Generic Formative Research of an Implementation Preparation
Source: JMIR Res Protoc. 2025 Sep 8;14:e69808. doi: 10.2196/69808 (PMC12455161; doi:10.2196/69808)
Supplement: Multimedia Appendix 7 [file resprot_v14i1e69808_app7.docx]

# **Optimising availability and appropriate use of assisted vaginal birth: a generic formative research protocol for implementation preparation**

##

## **Overview**

**As people who received the procedure, women play an important role in the uptake of assisted vaginal birth. When women are not aware of the procedure, women may feel scared, feeling left out of the decision-making process, and consequently may reject the procedure no due to negative interactions with providers, poor communication and mistrust of providers and systems. Some women may also fear pain and reject the procedure due to insufficient and unavailability of pain relief.**

**Information sharing with women about assisted vaginal birth is therefore pivotal. By sharing information with women, women are equipped with the necessary tools and information to participate in their care, make an informed choice, negotiate, and even actively make a decision together with the providers. This fosters empowerment and participation in their care to ensure that they are able to receive the best care possible. Unfortunately, to date, no intervention has been implemented incorporating women’s involvement.**

**Additionally, including family and community members in formative research on assisted vaginal birth is crucial because they significantly influence pregnant women's decisions in many cultural contexts. Understanding the perspectives of these key influencers, such as spouses, mothers-in-law, traditional birth attendants, and community leaders, allows for the development of targeted, culturally sensitive interventions that address specific concerns and misconceptions.**

**This primary qualitative research aims to understand women’s, family, and community members’ views, decision-making process, and acceptability of information sharing on assisted vaginal birth. We will explore what women, family, and community think about assisted vaginal birth, understand the needs and preferences of information sharing, and the best strategies to implement and disseminate information-sharing strategies to women. This comprehensive approach ensures that strategies are more relevant, widely accepted, and ethically inclusive, fostering community buy-in and improving the likelihood of successful implementation and sustainability of interventions aimed at promoting assisted vaginal birth.**

## **Target participants**

| Data collection methods and participants | | |
| --- | --- | --- |
| Population | **In-depth interview (IDI)** | **Focus group discussion (FGD)** |
| Care users |  |  |
| Postpartum women in the past 6 months regardless of mode of birth |  | **🗸** |
| Postpartum women in the past 6 months who experienced assisted vaginal birth | **🗸** |  |
| Women’s representatives who work in women’s organisations or associations | **🗸** |  |
| Family and community members |  |  |
| Partners |  | **🗸** |
| Mother in law |  | **🗸** |
| Community members (traditional birth attendant, queen mother, village champion, elderly women, or others; depending on local contexts) |  | **🗸** |

## **Resources and estimated time required to complete this module**

- Trained research assistants
- Audio recorders and notebooks for field notes
- Informed consent forms
- Private room for interview
- Focus group discussions with women: 60 to 90 minutes
- Interviews with women: 45 to 60 minutes

## **Focus group discussion guide with postpartum women regardless of the mode of birth**

***The sub-questions below (1a, 2b, 3c..) serve only as the probe to core/main questions (1, 2, 3..).***

*Interviewer: This section of the discussion is about what women know and how women perceived assisted vaginal birth. I would like to ask you some questions about what women in your community think about assisted vaginal birth.*

1. We would like to hear a little bit about your birth experience. Can you tell me how did you give birth to your baby?
2. How many times have you given birth in total, and were they vaginal births or caesarean sections?
3. In your community, do women give birth through other method, other than caesarean section or vaginal birth? Have you heard about assisted vaginal birth before?
   1. How did you come to know about assisted vaginal birth?
   2. Can you tell me a little bit about assisted vaginal birth? And how do you feel about it?
4. Thinking back to your antenatal care visits, have your providers ever explained to you about assisted vaginal birth before your childbirth?
   1. If women have been explained/aware about assisted vaginal birth: Can you tell me what they said?
   2. If women have not been explained/not aware about assisted vaginal birth: [Facilitators explaining assisted vaginal birth using assisted vaginal birth comic designed by the research team and letting participants read it]
      1. After reading about this comic, do you understand? Do you have any questions? What do you feel about assisted vaginal birth?
      2. What do you think about women receiving assisted vaginal birth to women instead of other options (i.e., caesarean section)? Do you think it will be beneficial or risky to women? Why and why not?

*Interviewer: This section of the discussion is about sharing information about assisted vaginal birth with women. I would like to ask you some questions about how you think we can appropriately share the information on assisted vaginal birth with women.*

1. Thinking about women in your community, do you think women in your community would like to get more information about assisted vaginal birth before their birth? Why or why not?
   1. At what point during pregnancy do you think women would want to receive information about assisted vaginal birth? Why?
   2. How do you think women in your community want to receive information about assisted vaginal birth?
      1. Would they want to receive this information verbally, from a healthcare provider? Why or why not?
      2. Would they want to receive this information in a comic like the want you read? Why or why not?
      3. Would they want to receive this information in a pamphlet or brochure? Why or why not?
      4. Would they want to receive this information using a computer or a mobile phone application? Why or why not?
   3. What type of information do you think women would want to know about assisted vaginal birth?
   4. Do you think women will be interested in seeing and touching assisted vaginal birth instruments at one point in their antenatal care when healthcare providers explaining about the procedure? Why or why not?
2. As a woman, how do you think women want to make decisions regarding assisted vaginal birth if the need arise/clinically indicated?
   - 1. Would women want to be involved in decision-making to have assisted vaginal birth during birth? Why or why not?
     2. How should providers involve women in decision-making? How can providers best support women regarding assisted vaginal birth?
     3. Would women want to discuss this information with other women, or their families before birth happen? Why or why not?
3. Do you have any other comments or feedback about assisted vaginal birth?

## **Interview discussion guide with postpartum women who experienced assisted vaginal birth**

***The sub-questions below (1a, 2b, 3c..) serve only as the probe to core/main questions (1, 2, 3..).***

*Interviewer: The purpose of this interview is to understand your preferences, experiences, and perceptions on assisted vaginal birth. Today, I would like to ask you about your birth experience, including what you know and how you think about assisted vaginal birth.*

1. I would like to hear a little bit about your birth experience. How many times have you given birth in total, and were they vaginal births or caesarean sections?
2. Can you tell me how did you give birth to your baby? How did your labour and birth go?
   1. Can you tell me how your labour start – did you go into labour on your own or was there an induction to help?
   2. When did your labour start? Is it before arrival to health facilities or at health facilities?
   3. How did you end up with assisted vaginal birth?
3. Before your birth, I am wondering if you already know about assisted vaginal birth?
   1. How did you come to know about assisted vaginal birth? When did you hear about this (was it before or after birth) and who told you?
   2. Can you tell me a little bit about what you know about assisted vaginal birth? And how do you feel about this procedure?
4. Thinking back to your antenatal care visits, have your providers ever explained to you about assisted vaginal birth before your childbirth? Can you tell me what they said?
5. Looking back, do you remember what type of instrument was used to assist your labour? How do you feel about it?
   1. What did the providers explain to you about this instrument? Were you given the option to choose other methods?
   2. How did the providers ask for your permission before conducting the procedure?
6. Looking back to your birth experience, were you aware of what was happening to you (your specific situation/condition at that time that you ended up needing assisted vaginal birth)? What explanation was given to you regarding your specific situation/condition?
7. Looking back to your birth experience, were you aware that assisted vaginal birth was used during the labour? What explanation was given to you regarding the use of assisted vaginal birth for you?
   1. When you were receiving the procedure, do you feel you have enough information about assisted vaginal birth? Why and why not?
   2. How did you feel about the information given to you about this procedure? And do you feel you gave permission for them to happen?
8. Can you tell me about what you like or don’t like about your birth experience?
   1. Looking back, would you choose this same way to give birth for your next pregnancy or choose a caesarean section instead? Why or why not?
9. Overall, do you feel is there anything that can be improved on your birth experience? How do you think your birth experience can be improved?
   1. What kind of information would you wish to receive before you give birth?
   2. How did you wish the healthcare providers informed or treated you during birth?
   3. What support did you wish to receive during your birth?
10. As a woman who had experienced assisted vaginal birth, would you prefer to get more information about assisted vaginal birth before, during, or after your birth? Why?
    1. At what point during pregnancy would you like to receive information about assisted vaginal birth? Why?
    2. How do you want to receive information about assisted vaginal birth?
       1. Would you want to receive this information verbally, from a healthcare provider? Why or why not?
       2. Would you want to receive this information in a pamphlet or brochure? Why or why not?
       3. Would you want to receive this information using a computer or a mobile phone application? Why or why not?
    3. What type of information do you want to know about assisted vaginal birth?
11. Looking back, how do you think the decision-making process on assisted vaginal birth could be improved?
    - 1. Would you want to be involved in the decision-making of this procedure during birth? Why or why not?
      2. How should providers involve you in decision-making? How can providers best support you regarding assisted vaginal birth?
      3. Would you want to discuss this information with other women, or their families before birth happen? Why or why not?
12. Do you have any other comments, questions, or feedback in regard to your birth experience?

## **Interview discussion guide with women’s representatives who work in women’s organisations or associations**

***The sub-questions below (1a, 2b, 3c..) serve only as the probe to core/main questions (1, 2, 3..).***

*Interviewer: This section of the discussion is about the social norms and factors that are important to women in their communities. I would like to ask you some questions about what women you represent may think about assisted vaginal birth.*

1. In your community, do women give birth through other method, other than caesarean section or vaginal birth? Have you heard about assisted vaginal birth before?
   1. How did you come to know about assisted vaginal birth?
   2. Can you tell me a little bit about assisted vaginal birth? And how do you feel about it?
2. [Facilitators explaining assisted vaginal birth using assisted vaginal birth comic designed by the research team and letting participants read it]
   1. After reading about this comic, what do you think the women you represent may feel about assisted vaginal birth?
   2. What do you think about women you represent receiving assisted vaginal birth instead of other options (i.e., caesarean section)? Do you think it will be beneficial or risky to women? Why and why not?
3. In the women’s community that you represent, do you think the women whom you represent are aware of assisted vaginal birth?
   1. How do the women you represent often come to know about assisted vaginal birth?
   2. Can you tell me what women you represent may know about assisted vaginal birth? And how may they feel about it?
4. Thinking about antenatal care visits, do you think providers ever or should explain to women you represent about assisted vaginal birth before their childbirth? Can you tell me what they may/should explain to the women you represent?

*Interviewer: This section of the discussion is about sharing information about assisted vaginal birth with women. I would like to ask you some questions about how you think we can appropriately share the information on assisted vaginal birth with the women you represent.*

1. Thinking about the women you represent; do you think women would like to get more information about assisted vaginal birth before their birth? Why or why not?
   1. At what point during pregnancy do you think women you represent would want to receive information about assisted vaginal birth? Why?
   2. How do you think the women you represent want to receive information about assisted vaginal birth?
      1. Would they want to receive this information verbally, from a healthcare provider? Why or why not?
      2. Would they want to receive this information in a comic like the want you read? Why or why not?
      3. Would they want to receive this information in a pamphlet or brochure? Why or why not?
      4. Would they want to receive this information using a computer or a mobile phone application? Why or why not?
   3. What type of information do you think women you represent would want to know about assisted vaginal birth?
   4. Do you think the women you represent will be interested in seeing and touching assisted vaginal birth instruments at one point in their antenatal care when healthcare providers explaining about the procedure? Why or why not?
2. As a woman’s representative, how do you think women want to make decisions regarding assisted vaginal birth?
   - 1. Would women want to be involved in decision-making to have assisted vaginal birth during birth? Why or why not?
     2. How should providers involve women in decision-making? How can providers best support women regarding assisted vaginal birth?
     3. Would women want to discuss this information with other women, or their families before making the decision before the birth happen? Why or why not?
3. Do you have any other comments or feedback about assisted vaginal birth?

## **Focus group discussion guide with women’s partner, family members, and community members**

***The sub-questions below (1a, 2b, 3c..) serve only as the probe to core/main questions (1, 2, 3..).***

*Interviewer: This section of the discussion is about the social norms and factors that are important to women in their communities. I would like to ask you some questions about what your family and community may think about assisted vaginal birth.*

1. In your community, do women give birth through other method, other than caesarean section or vaginal birth? Have you heard about assisted vaginal birth before?
   1. How did you come to know about assisted vaginal birth?
   2. Can you tell me a little bit about assisted vaginal birth? And how do you feel about it?
2. [Facilitators explaining assisted vaginal birth using assisted vaginal birth comic designed by the research team and letting participants read it]
   1. After reading about this comic, what do you think the women in your community may feel about assisted vaginal birth?
   2. What do you think about women in your community receiving assisted vaginal birth instead of other options (i.e., caesarean section)? Do you think it will be beneficial or risky to women? Why and why not?
3. In your community, do you think the women are aware of assisted vaginal birth?
   1. How do the women in your community often come to know about assisted vaginal birth?
   2. Can you tell me what women in your community may know about assisted vaginal birth? And how may they feel about it?
4. Thinking about antenatal care visits, do you think providers ever or should explain to women in your community about assisted vaginal birth before their childbirth? Can you tell me what they may/should explain to the women in your community?

*Interviewer: This section of the discussion is about sharing information about assisted vaginal birth with women. I would like to ask you some questions about how you think we can appropriately share the information on assisted vaginal birth with the women* in your community*.*

1. Thinking about the women in your community; do you think women would like to get more information about assisted vaginal birth before their birth? Why or why not?
   1. At what point during pregnancy do you think women in your community would want to receive information about assisted vaginal birth? Why?
   2. How do you think the women in your community want to receive information about assisted vaginal birth?
      1. Would they want to receive this information verbally, from a healthcare provider? Why or why not?
      2. Would they want to receive this information in a comic like the want you read? Why or why not?
      3. Would they want to receive this information in a pamphlet or brochure? Why or why not?
      4. Would they want to receive this information using a computer or a mobile phone application? Why or why not?
   3. What type of information do you think women in your community would want to know about assisted vaginal birth?
   4. Do you think the women in your community will be interested in seeing and touching assisted vaginal birth instruments at one point in their antenatal care when healthcare providers explaining about the procedure? Why or why not?
2. How do you think women in your community want to make decisions regarding assisted vaginal birth?
   - 1. Would women in your community want to be involved in decision-making to have assisted vaginal birth during birth? Why or why not?
     2. How should providers involve women in decision-making? How can providers best support women regarding assisted vaginal birth?
     3. Would women want to discuss this information with other women, or their families before making the decision before the birth happen? Why or why not?
3. Do you have any other comments or feedback about assisted vaginal birth?
