# Supplementary material for: Optimizing Availability and Appropriate Use of Assisted Vaginal Birth: Protocol for Generic Formative Research of an Implementation Preparation
Source: JMIR Res Protoc. 2025 Sep 8;14:e69808. doi: 10.2196/69808 (PMC12455161; doi:10.2196/69808)
Supplement: Multimedia Appendix 8 [file resprot_v14i1e69808_app8.docx]

# **Optimising availability and appropriate use of assisted vaginal birth: a generic formative research protocol for implementation preparation**

## **Overview**

**The main barrier to performing assisted vaginal birth among providers is resistance to change due to beliefs surrounding assisted vaginal birth. Studies have shown that healthcare providers often hesitate in conducting assisted vaginal birth as they perceived the procedure as complicated and dangerous. Providers also feel not confident in conducting the procedure as they feel they lack skills and supervision and fear that there will be litigation if complications occur, and fear HIV transmission. Furthermore, the use of assisted vaginal birth could be discouraged by an unsupportive work environment where training is not delivered to learn and practice assisted vaginal birth (including when to stop and proceed to caesarean), lack of mentoring, poor communication, power struggles, the culture of blaming, lack of clarity scope of tasks and functions, and lack of equipment including pain relief and sterilisation.**

**There is an emphasis on training provision and sustaining the knowledge and skills of providers surrounding assisted vaginal birth. Torloni et al (2023) reported the provision of equipment (including pain relief for women), implementation of learning (didactic training through lecture or theoretical class, simulation training, practical hands-on training, and onsite hands-on training), implementation of assisted vaginal birth guidelines, and audit and feedback for quality assurance are most implemented to optimise assisted vaginal birth. Furthermore, WHO technical consultation on assisted vaginal birth highlighted the need on addressing fear of litigation, blaming culture, and possibilities of task-shifting implementation to optimise use.**

Information on the effectiveness, acceptability, and how to appropriately implement these potential interventions remains unknown. **Therefore, this primary qualitative research aims to understand providers’ views, decision-making process, and acceptability of potential interventions in optimising assisted vaginal birth use. We will explore what providers and administrators’ think about assisted vaginal birth, understand their acceptability of different potential interventions, and the best strategies to implement the interventions.**

## **Participants in qualitative research**

| Data collection methods and participants | | |
| --- | --- | --- |
| Population | **In-depth interview (IDI)** | **Focus group discussion (FGD)** |
| Care providers |  |  |
| Doctors | **🗸** |  |
| Midwives and/or nurses | **🗸** |  |
| Administrators (e.g., matron in charge) | **🗸** |  |

## **Resources and estimated time required to complete this module**

- Trained research assistants
- Audio recorders and notebooks for field notes
- Informed consent forms
- Private room for interview
- Interviews with healthcare providers and administrators: 1 to 2 hours

## **Interview guide for providers and administrators**

***The sub-questions below (1a, 2b, 3c..) serve only as the probe to core/main questions (1, 2, 3..).***

*Interviewer: The purpose of this interview is to understand preferences and experiences on assisted vaginal birth from the perspectives of healthcare providers and administrators. Today, I would like to ask you what you think about the procedure and how its use can be optimised.*

#### Decision-making on assisted vaginal birth

1. I would like to know more a little bit about your roles here. Can you tell me a bit about your work here in [facility]? How many years have you worked here?
2. Have you heard about assisted vaginal birth before? Can you tell me about your experiences in conducting assisted vaginal birth?
   1. Which instruments do you use more than the other (vacuum vs forceps)? Do you have any preference between forceps and vacuum? Why?
   2. How often per month do you think you have performed assisted vaginal birth in your facility?
   3. Where did you learn about performing assisted vaginal birth? Probe around clinical training or in-service training
   4. How do you think assisted vaginal birth use in facility? Is it used often? Why or why not?
3. [if provider/facility conduct assisted vaginal birth] Can you tell me how women are typically indicated to need assisted vaginal birth in your health facility?
   1. How and when do you normally decide a woman should have assisted vaginal birth? How do you normally inform and engage women to make shared decision-making?
   2. At what point do you normally stop trying to go for assisted vaginal birth, and proceed to caesarean section?
   3. In your facility, do you have any guidelines in regards to assisted vaginal birth use? If yes, what does it says? If not, what guidelines do refer to in regards to assisted vaginal birth? Why or why not?
4. In your opinion, what are some of the gaps or barriers or challenges that influence your decision not to use assisted vaginal birth despite being able to conducted? [If the hospital does not offer assisted vaginal birth, this question should be rephrased in a hypothetical way]
   1. Explore other barriers that are independent of training or availability of equipment

#### Provision of equipment

1. What equipment and other resources do you need to enable you in performing assisted vaginal birth?
   1. How available are these equipment and resources in your facility?
2. What pain relief options are available for women undergoing assisted vaginal birth at your facility?
   1. How frequently do you normally offer/administer pain relief to women undergoing assisted vaginal birth? How do you do this and when?

#### Learning and training components

1. Does your facility provide in-service training for labour management, including assisted vaginal birth?
   1. If yes, can you tell me how the training was conducted and what was covered? Is it once or multiple times throughout your service time?
   2. In your opinion, how useful are the training(s)? What could be done to make them more useful?
2. In your opinion, what are some of the gaps in training about performing assisted vaginal birth among health workers working in your facility? Any differences between midwives and doctors?
3. Imagine that your facility is going to deliver training on labour management, including assisted vaginal birth? How do you expect the training would be implemented?
   1. What should be covered?
   2. How should it be delivered (didactic training, simulation training, practical hands-on training, and onsite, hands-on supervision, other)?
   3. When should it be delivered?
   4. How often should it be delivered?
   5. Who should receive it?
4. How can health care providers maintain their skills and confidence after the training?

#### Assisted vaginal birth guidelines

1. Does your facility have any clinical guidelines or protocols in relation to labour management, including performing assisted vaginal birth? If yes, can you tell me more about them?
   1. In your opinion, how useful are these clinical guidelines or protocols? What could be done to make them more useful?

#### Audit and feedback

1. In terms of monitoring and reporting, can you tell me how your facility monitors the use of assisted vaginal birth and its relevant outcomes? Has this ever been implemented before?
2. Have you heard about audit and feedback implementation before? What do you think about implementing audit and feedback on assisted vaginal birth and its relevant outcomes?
3. Imagine that your institution is planning to implement audit and feedback for audit and feedback use, how do you think it should be implemented?
   1. What indicators should be reported?
   2. How do you think it should be conducted?
   3. How often should it be conducted?
   4. Who should be involved in the audit and feedback?
   5. What should be done to prepare for and sustain this?

#### Opinion leaders

1. Have you heard about local champions before? What do you think about appointing a local champion to promote the optimised use of assisted vaginal birth at your facility?
2. If your institution is going to implement a local champion for an assisted vaginal birth, how do you think the local champion should be selected?
   1. Who do you think it should be?
   2. What do you expect the local champion would do?
   3. What kind of interaction do you want the local champion to have with you?

#### Non-technical skills (i.e., communication, supporting women, joint decision-making)

1. During antenatal care, do you normally mention and discuss with women about assisted vaginal birth?
   1. If yes, can you tell me how this normally happens?
   2. If no, what are the reasons that you consider deciding not to discuss this with woman?
2. In your opinion, what information do you think could be included in prenatal education about assisted vaginal birth?
   1. Do you think women would want to have information about:
      1. What to expect during a vaginal birth? Why or why not?
      2. Indications of assisted vaginal birth?
      3. Risks of assisted vaginal birth? Why or why not?
      4. Benefits of assisted vaginal birth? Why or why not?
   2. Are there additional aspects of assisted vaginal birth that women would like to know about? How do you think women would feel about receiving information on the risks and benefits of an alternative method, such as a second-stage cesarean section?
   3. At what point during a woman’s pregnancy do you think they should receive this information about assisted vaginal birth? Why?
   4. How often do you think women should receive information about assisted vaginal birth?
   5. How would you think women should receive this information?
      1. Should she receive this information verbally, from her healthcare provider? Why or why not?
      2. Should she receive this information in a pamphlet or brochure? Why or why not?
      3. Should she receive this information using a computer or a mobile phone application? Why or why not?
3. Do you have any other comments or feedback on using assisted vaginal birth?
