# Supplementary material for: Optimizing Availability and Appropriate Use of Assisted Vaginal Birth: Protocol for Generic Formative Research of an Implementation Preparation
Source: JMIR Res Protoc. 2025 Sep 8;14:e69808. doi: 10.2196/69808 (PMC12455161; doi:10.2196/69808)
Supplement: Multimedia Appendix 9 [file resprot_v14i1e69808_app9.docx]

# **Understanding policymakers’ views on assisted vaginal birth and acceptability on strategies to sustain interventions to optimize assisted vaginal birth use**

## **Overview**

Involving policymakers in efforts to optimise assisted vaginal birth use is crucial to ensuring consistent messages to support and training from international and national bodies and to ensure financing for the sustainability of the interventions. Based on WHO technical consultation, the focus on an interview with policymakers will include:

- Facilitating assisted vaginal birth in primary healthcare settings, including continuity on the provision of training and equipment
- Funding to acquire, replace, and maintain essential equipment including instruments needed
- Implementation of a local champion for assisted vaginal birth
- Implementation of regular local/regional audits and feedback on assisted vaginal birth alongside core maternal and perinatal outcomes
- Exploring the creation or adaptation of national guidelines on AVB
- Explore the possibility on including assisted vaginal birth training in the formal curricula of medical schools, residency programs, nursing, and midwifery schools
- Advocacy strategy to engage policymakers in encouraging evidence-based practices more effectively

The policymakers here could be the Ministry of Health, local health offices, or international funders in the country, involved in maternal health work, and driving changes and policies around birth management. The aim of this primary research is to understand policymakers’ views on assisted vaginal birth and their acceptability of strategies to sustain interventions to optimise assisted vaginal birth use.

## **Participants in qualitative research**

| Data collection methods and participants | | |
| --- | --- | --- |
| Population | **In-depth interview (IDI)** | **Focus group discussion (FGD)** |
| Policymakers | **🗸** |  |

## **Resources and estimated time required to complete this module**

- Trained research assistants
- Audio recorders and notebooks for field notes
- Informed consent forms
- Private room for interview
- Interviews with healthcare providers and administrators: 45 minutes to 1 hour

## **Interview guide for policymakers**

***The sub-questions below (1a, 2b, 3c..) serve only as the probe to core/main questions (1, 2, 3..).***

*Interviewer: The purpose of this interview is to understand the institution’s priorities on assisted vaginal birth from the perspectives of policymakers like you. Today, I would like to ask you what you think about assisted vaginal birth and potential strategies to sustain it.*

1. I would like to know more a little bit about your roles here. Can you tell me a bit about your work here in [institution]? How long have you been working here?
2. Have you heard of assisted vaginal birth before? If yes, can you tell me your thoughts about assisted vaginal birth?

#### Facilitating assisted vaginal birth in primary healthcare settings

1. How feasible do you think it would be to implement assisted vaginal birth in primary healthcare settings?
   1. What are the challenges that might be faced to implement assisted vaginal birth in primary healthcare settings?
   2. What do you think might help to implement assisted vaginal birth in primary healthcare settings?

#### Funding to acquire, replace, and maintain essential equipment including instruments needed

1. What type of resources do you think you would need to implement assisted vaginal birth in different types of health facilities?
   1. What type of financial resources would you need to implement assisted vaginal birth?
   2. What equipment would you need to implement assisted vaginal birth?
   3. How available are these financial, equipment and other resources?
   4. How do you think a continuous supply of these resources can be ensured to sustain assisted vaginal birth implementation?

#### Implementation of regular local/regional audits and feedback on assisted vaginal birth alongside core maternal and perinatal outcomes

1. In terms of monitoring and reporting, can you tell me how your institution monitors the use of assisted vaginal birth from facilities and its relevant outcomes? Has this ever been implemented before?
2. Have you heard about audit and feedback implementation before? What do you think about implementing audit and feedback on assisted vaginal birth and its relevant outcomes? Will this be beneficial for your institution?
3. Imagine that your institution is receiving audit and feedback data from health facilities, what kind of data would you like to receive?
   1. What indicators should be reported?
   2. How often do you think it should be reported?
   3. What should be done to prepare for and sustain this?

#### Assisted vaginal birth guidelines

1. Does your institution have any clinical guidelines or protocols on assisted vaginal birth use that you issue or recommend for use?
   1. If yes, can you please tell me more about them? In your opinion, how useful are these clinical guidelines or protocols? What could be done to make them more useful?
   2. If no, how feasible do you think to issue or include assisted vaginal birth use to current guidelines on labour management?
      1. What should it take to create and adapt, and how do you think it should be disseminated?

#### Explore the possibility on including assisted vaginal birth training in the formal curricula of medical schools, residency programs, nursing, and midwifery schools

1. Do you know if assisted vaginal birth training is included in training in the formal curricula of medical schools, residency programs, nursing, and midwifery schools?
   1. If yes, can you tell me what you know about this? What are some of the gaps in training on assisted vaginal birth?
   2. If no, how feasible do you think to include and implement assisted vaginal birth training in the formal curricula of medical schools, residency programs, nursing, and midwifery schools?
      1. What information would be helpful to be included in the curricula?
      2. What should it take to implement this?

#### Advocacy strategy to engage policymakers in encouraging evidence-based practices more effectively

1. How involved do you think policymakers are in encouraging assisted vaginal birth use in your area?
2. What do you think is the best advocacy strategy for engaging policymakers and encouraging evidence-based practices more effectively?
3. Do you have any other comments or feedback about assisted vaginal birth?
