# Supplementary material for: Optimizing Availability and Appropriate Use of Assisted Vaginal Birth: Protocol for Generic Formative Research of an Implementation Preparation
Source: JMIR Res Protoc. 2025 Sep 8;14:e69808. doi: 10.2196/69808 (PMC12455161; doi:10.2196/69808)
Supplement: Multimedia Appendix 11 [file resprot_v14i1e69808_app11.pdf]

FOR WHO/HRP USE ONLY

Date submitted to RP2

Thematic area:

☐ Single site proposal

☐ "Core" proposal (for multicenter study)

☒ Generic protocol

☐ Centre-specific proposal under multicenter study

☐ Multi-site/multi-country proposal

☐ Phase I (or) ☐ Phase II of new project

☐ Amendment

☐ Continuing review

Connect ID No:

Project title

Optimising availability and appropriate use of assisted vaginal birth: a generic formative research protocol for implementation preparation

Type of review: New generic formative research protocol

Project/TRIMS ID: A66054

Protocol version and date (*mandatory information*): version 3 dated 22 January 2024

REVIEWERS NAMES: Eileen Yam and Natalie Hammond

RESPONSIBLE OFFICER: Ana Pilar Betrán

PRINCIPAL INVESTIGATOR: Meghan A Bohren

RP2 assessment

The *proposal* of generic protocol: **Has been APPROVED**

There is no budget associated with this genetic protocol.

Duration of project: 1-year, tentative January 2024- December 2024

Date of approval: 02 February 2024

Review comments and answers are found below.

Review approval prepared by (PS)

Secretariat of the Reseearch Project Review Panel (RP2) I Department of Sexual and Reproductive Health and Research I Human reproduction programme (HRP)

RP2@who.int
