# Supplementary material for: Optimizing Availability and Appropriate Use of Assisted Vaginal Birth: Protocol for Generic Formative Research of an Implementation Preparation
Source: JMIR Res Protoc. 2025 Sep 8;14:e69808. doi: 10.2196/69808 (PMC12455161; doi:10.2196/69808)
Supplement: Multimedia Appendix 10 [file resprot_v14i1e69808_app10.pdf]

20, AVENUE APPIA – CH-1211 GENEVA 27 – SWITZERLAND – HTTP://WWW.WHO.INT/ETHICS/REVIEW-COMMITTEE/EN/ – HTTPS://EXTRANET.WHO.INT/ERCWEB/LOGIN.PHP

WHO ERC
Review Summary

Protocol ID: A66054
Country: N/A Template
Protocol Title: Optimising availability and appropriate use of assisted vaginal birth: a generic formative research protocol for implementation preparation
Version: 4 **Dated:** 19.04.2024 **Submitted:** 07.05.2024
WHO Responsible Staff Member: Ana Pilar Betran Lazaga
Responsible Unit: WHO/HQ/FWC/RHR
Meeting Date: 13.06.2024

Dear Dr. Betran Lazaga,
Please find the review summary of the Protocol "Optimising availability and appropriate use of assisted vaginal birth: a generic formative research protocol for implementation preparation", which was first submitted to the Secretariat on 26.02.2024. This proposal underwent regular review.

The outcome of the review is provided below. When responding, please submit the following:

1. A cover memorandum that addresses your responses, POINT BY POINT, to each of the queries in sections A and B.
   Section C contains Suggestions to improve the proposal but there is no obligation to follow them.

2. An Amended protocol including the responses in bold, highlighted or in track changes. Please ensure that tracking formatting changes is switched off or that all formatting changes have been accepted and that no comments which the team may exchange during the editing are included in the track changes version. The protocol should include all relevant documentation (ICF, study instruments, peer review, etc.) even if already submitted.

Please note that comments in the introductory paragraph are meant for the WHO Responsible Staff Member, though you may decide to share them with the PI.

PLEASE RESPOND TO THIS REVIEW SUMMARY WITHIN A 3 MONTH PERIOD, OR PROVIDE THE ERC SECRETARIAT A VALID JUSTIFICATION FOR THE DELAY.

The ERC considered this submission a generic template protocol. The aim of this tool is to support Member States and other stakeholders to develop site-specific protocols for formative research in assisted vaginal birth (AVB) that will inform the design and implementation of health interventions. The ERC recognised the high potential social value of such a tool.

Recognising that the template protocol will be shared freely, and that end users of the tool may or may not conduct the resulting research with WHO support, whether technical or financial, meaning these studies may not be under WHO (or WHO ERC) oversight. In creating this tool, WHO has a responsibility to ensure that the guidance within is robust from an ethical perspective, so that the ethical considerations raised by this kind of research are easier for end users to manage and to better support the interests of future participants in these studies. The ERC identified issues regarding participants' privacy, confidentiality, and personal data as key concerns which much be clearly addressed in this generic master to ensure the document is appropriately applied in diverse country contexts.

Once this template is approved, please note that any site-specific protocols based on the approved template and that will be implemented with WHO support are to be submitted to the ERC for review and approval. Specific comments to address are outlined below.

A. Amendments (Response and change required)

This section includes queries and comments on your protocol, study instruments or the informed consent form for which the ERC requires your response and where relevant, appropriate amendments to the protocol, study instruments or the informed consent.

1. Protocol

1.1. Please provide an amended proposal specifying the version number and/or date on each page.

1.2. Respect for persons:

1.2.1. The readiness assessment involves direct observation of maternity wards and discussion with staff. In responding to a request from the Secretariat for clarification on how consent will be obtained for the observations from health workers and patients, the investigators stated there will be no interaction or consultation with patients during the readiness assessment, and that the instrument states "Prior to conducting the readiness assessment, all maternity units in the facility should be informed and briefed on the purpose of the readiness assessment and how it will be conducted." However, further clarification is needed in the protocol.

1.2.1.1. Please clarify how the researchers will inform the facility managers, medical staff and patients of these activities and how their permission will be sought. The protocol should state explicitly that authorisation will be sought from the administration/management of health facilities.

1.2.1.2. Readiness assessment observations have the potential to raise the following concerns, and a detailed explanation is requested of how these risks will be mitigated: i) the assessment may interfere with the daily duties of the medical staff and routine medical practice; and ii) it may involve observation of interactions among the medical staff as well as between the staff and the patients and their family members, which may compromise the privacy and intimacy of the patients.

1.3. Risk/benefit considerations:

1.3.1. The study protocol describes the advantages of AVB but not its potential risks. It is important to also mention any potential risks of AVB, such as the risk of excessive bleeding or vaginal tears, etc, (and the chances of adverse occurrence, even if minimal) and describe how these risks may be mitigated or addressed. Please address this in the protocol and also in the participant information leaflet.

1.4. Data management: Researchers are encouraged to review the data management and storage procedures.

1.4.1. The protocol states that the IDIs and FGDs will be audio recorded, transcribed afterward, and the transcripts will be de-identified. The protocol (p. 18) states that the original audio-recorded data will be kept in separate folders from the de-identified data to avoid identification, and that all data will be kept securely in institutional storage for 5 years from the date of publication and may be deleted or destroyed after this time. The ERC identified three problems with these solutions:

1.4.1.1. There seems to be no scientific need or justification for keeping the original sound recordings after they have been transcribed. The audio recordings contain personal data and - especially in the case of IDIs and FGDs with women - concern very sensitive, intimate issues. In order to best protect the privacy of the participants, the audio reading should be deleted after the transcript has been prepared and verified. Please revise accordingly.

1.4.1.2. The 5-year data retention period seems arbitrary and poorly defined. It is unclear what publication the "publication date" refers to (e.g., the first conference presentation of study results, the date of publication of the first written preliminary report, the date of the last study-related scientific article), whereas study completion would be a clearer point of reference for data storage. As this is a generic template protocol, please state that data protection laws and regulations in each country and each site (since individual clinics might have their own procedures in place) will be adhered to, and data storage must comply with local data governance/policies and ethics committee requirements. Ideally the specification of the retention period would be left to the requirements of the study sites and stakeholders rather than being prescriptive with a particular number of years. .

1.4.1.3. In the protocol and consent documents, the recommended storage time should be clear. The information that all research data "may be deleted or destroyed after that time [i.e., retention period]" is ambiguous. Instead of saying that the data "may be deleted", please state when the data will be deleted.

1.4.2. Please ensure the protocol provides clear guidance on important points to be specified by site-specific protocols such as: where data should be safely kept; for how long; who will have access to data (with

consideration to what will happen if key staff leave); when data will be destroyed; and any anticipated future uses and the mechanisms for deciding these.

1.5. Social value:

1.5.1. After the assessments and implementation of the protocol, it is somewhat unclear what institutions should do if they identify a problem. It will be helpful to include some recommendations on steps to rectify or address challenges to ensure sites are ready to adopt or improve on AVB and uptake, where required. Please respond to this concern.

1.5.2. The protocol emphasises the autonomy of women in making decisions about their birthing choices, which is appropriate. However, in some contexts where birthing choices are likely to be influenced by close relations, such as the spouse or mother-in-law, if their views about AVB are clearly known, they could then be used to inform targeted interventions in places where these individuals may influence a woman's decision to accept AVB or not. For this reason, please consider including these allied decision makers as key stakeholders in the research.

1.5.3. The protocol already mentions other groups and includes men/husbands as an add-on, but it would be important to have another core component for interview groups for other family or community members whose advice and decisions may directly affect the pregnant women and their decisions in some contexts (e.g. traditional birth attendant, queen mother, village champion, or elderly women in the community.

1.6. Dissemination: Under Data Analyses (p. 17), the protocol states that participants will receive a study report summarising research findings. Please also consider recommending that findings be shared with community leaders to be disseminated more broadly within communities.

2. Study Instruments

2.1. Questionnaire: Some questions may be sensitive to ask and do not seem immediately related to the objectives of the protocol. For example, questions about personal experiences of stillbirths and miscarriages. If these questions really do need to be asked, please explain the rationale for asking these sensitive questions in terms of the scientific objectives of the study, and why this information may not be ascertained through other means (e.g., document review).

2.2. The in-depth interview questions are quite numerous due to all the sub-questions. Because this is a generic master and may be implemented in quite diverse settings, please consider recommending a minimum set of core questions that will be helpful to address the objectives of the study (with least time and burden to the participants), and those that could be asked as additional questions if time allows or if a site would like to do further sub-analysis.

2.3. Comic images: It would be useful to include a line in the protocol to indicate that teams should check (e.g., with a community advisory board) whether the images are culturally appropriate or that they should be adapted or replaced with culturally acceptable material.

3. Informed Consent Forms

3.1. The consent documents are written in a fairly sophisticated language in terms of vocabulary and structure, especially the parts concerning data management and storage. Expressions such as "audio-recorded data", "de-identified data", etc., should be replaced by more reader-friendly ones. Please review the consent documents to ensure these are simple and easily understood by a layperson, particularly those for women and women's organisations.

3.2. The participant information sheet describes the kind of questions that will be covered in the interviews, but not what will be asked of participants in focus group discussions. Please clarify for participants.

3.3. The protocol specifies that interviews and FGDs will be recorded, by the consent documents indicate they *may* be recorded. Please clarify and revise for consistency.

B. Clarifications (Response required but change may not be required)

NIL

C. Suggestions

This section consists of suggestions for alternative scientific or technical approaches or methods for conducting the research but which do not raise critical, ethical issues. These are meant to be helpful to investigators and are presented as suggestions for you to consider incorporating into a revised protocol. No response from you is required for any comment in this section. If, however, you do make changes to the protocol as a result of these suggestions, please submit the revised protocol to the ERC.

1. Study Instruments
   1.1. Some of the questions proposed are quite straightforward to answer and researchers could consider asking these in a survey form and to a broader participant pool rather than including all questions in the in-depth interviews.
   1.2. It would be helpful if the protocol could include a Q&A about AVB for women or health providers.

Noticed in the review and highlighted here to assist the study team only:

- Page 35: This is done to preserve any linguistic nuances that could be *loss* [replace with “lost”] during the translation process.

Protocol ID: A66054 **Meeting Date**: 13.06.2024

Based on the above comments, the Committee has the following recommendation(s) for this proposal:

[ ] The proposal is ***Approved as submitted.*** No modifications are required.

[X] The proposal is ***Conditionally Approved; requires amendments and/or clarifications.*** Final approval is contingent upon an adequate response by the Principal Investigator, to the satisfaction of the reviewers or the Chair on behalf of the ERC.

[ ] The proposal is ***Not approved; requires additional information and/or rewriting.*** A revised version of the proposal should be re-submitted by the WHO responsible staff member as a new submission to the ERC for re-review by Committee.

[ ] The proposal is ***Rejected***. The proposal is ethically unacceptable, for the reasons stated above. The Principal Investigator may submit a new proposal that takes into consideration the ethical issues raised by the Committee. If you do not agree with the Committee's assessment, please feel free to submit an appeal to the Chair of the ERC, through the Secretariat.

NOTE: Final Approval of the Proposal is contingent upon submission of the following:

[ ] Local ethics approval(s) [ ] Other relevant documents

The ERC would like to receive a copy of the recommendations of the local ethics committee when available.

IMPORTANT

1. *Any changes to the proposal or to the attachments (informed consent/study instruments etc.) should be approved by the ERC before being implemented.*
2. *The approval for this proposal is valid for a period of* ***one year only.***
3. *Please resubmit this proposal for a Continuing Review at least 2 months before the next re-approval period.*

Chairperson Ehsan Shamsi Gooshki **Date 28/06/2024**

Name: Ehsan Shamsi Gooshki

FINAL APPROVAL	FOR THE SECRETARIAT
Amendments and Clarifications to the proposal have been reviewed.<br>The protocol (Version: 5 Dated: 16.07.2024) submitted on 20.08.2024 and informed consent forms (Version: 6 Dated: 05.09.2024) submitted on 05.09.2024 are approved by the ERC.<br>N.B.: Any site-specific studies based on this ERC-approved template that are implemented with WHO technical or financial support must be submitted to the WHO ERC for review and approval prior to implementation.<br><br>**Chairperson** Ehsan Shamsi Gooshki<br>**Name** **Ehsan Shamsi Gooshki**<br>**Date** **05/09/2024**	Amendments and Clarifications to be reviewed:<br>[ ] Electronically by ERC<br>[ ] by Primary reviewers<br>[ ] by Secretariat<br><br>Amendments approved / Clarifications accepted on 5.9.2024<br>Local ERC approval(s) obtained on N/A (WHO template)<br>Relevant Documents submitted on<br><br>Comments:<br><br>Signature: L. Pascoe Date: 5.9.2024
